# The effects of nature‐inspired amino acid substitutions on structural and biochemical properties of the *E. coli* L‐asparaginase EcAIII

**DOI:** 10.1002/pro.4647

**Published:** 2023-06-01

**Authors:** Maciej Janicki, Anna Ściuk, Andrzej Zielezinski, Milosz Ruszkowski, Agnieszka Ludwików, Wojciech M. Karlowski, Mariusz Jaskolski, Joanna I. Loch

**Affiliations:** ^1^ Department of Biotechnology, Institute of Molecular Biology and Biotechnology, Faculty of Biology Adam Mickiewicz University Poznań Poland; ^2^ Department of Crystal Chemistry and Crystal Physics, Faculty of Chemistry Jagiellonian University Krakow Poland; ^3^ Department of Computational Biology, Faculty of Biology Adam Mickiewicz University Poznań Poland; ^4^ Institute of Bioorganic Chemistry Polish Academy of Sciences Poznań Poland; ^5^ Department of Crystallography, Faculty of Chemistry Adam Mickiewicz University Poznań Poland

**Keywords:** L‐asparaginase, ligand docking, mutagenesis, orthologs, protein structure

## Abstract

The *Escherichia coli* enzyme EcAIII catalyzes the hydrolysis of L‐Asn to L‐Asp and ammonia. Using a nature‐inspired mutagenesis approach, we designed and produced five new EcAIII variants (M200I, M200L, M200K, M200T, M200W). The modified proteins were characterized by spectroscopic and crystallographic methods. All new variants were enzymatically active, confirming that the applied mutagenesis procedure has been successful. The determined crystal structures revealed new conformational states of the EcAIII molecule carrying the M200W mutation and allowed a high‐resolution observation of an acyl‐enzyme intermediate with the M200L mutant. In addition, we performed structure prediction, substrate docking, and molecular dynamics simulations for 25 selected bacterial orthologs of EcAIII, to gain insights into how mutations at the M200 residue affect the active site and substrate binding mode. This comprehensive strategy, including both experimental and computational methods, can be used to guide further enzyme engineering and can be applied to the study of other proteins of medicinal or biotechnological importance.

**Abbreviations**
AI: artificial intelligence
IFD: induced fit docking
IPTG: isopropyl β‐D‐1‐thiogalactopyranoside
MD: molecular dynamics
MM‐GBSA: molecular mechanics‐generalized Born surface area
nanoDSF: differential scanning fluorimetry in the nano‐scale
NPT: constant‐temperature, constant‐pressure ensemble
OPLS: optimized potentials for liquid simulations
PDB: Protein Data Bank
rmsf: root‐mean‐square fluctuation
SP: standard precision
SSM: position‐specific scoring matrix
PVA: polyvinyl alcohol
rmsd: root‐mean‐square deviation
TCA: trichloroacetic acid
TIP3P: transferable intermolecular potential 3 point
VSGB: variable‐dielectric generalized Born

## 1 INTRODUCTION

L‐Asparaginases are enzymes that hydrolyze L‐asparagine to L‐aspartate and ammonia. L‐Asparaginases are divided into three structural Classes (da Silva et al., 2022; Loch & Jaskolski, 2021). L‐Asparaginases from Class 1, thanks to their high substrate affinity (μM), are used in the treatment of lymphoproliferative disorders (Apostolidis et al., 2020; Cachumba et al., 2016; Maggi et al., 2017; Muneer et al., 2020; Patel et al., 2022); some of them are also used in the food industry (Paul & Tiwary, 2020; Xu et al., 2016). Class 3 of L‐asparaginases includes *Rhizobium etli*‐type enzymes with an extraordinary catalytic center and (mM) substrate affinity (Loch et al., 2021; Moreno‐Enriquez et al., 2012). L‐Asparaginases of Class 2 belong to the Ntn‐hydrolases and have (mM) affinity for the substrate (Borek et al., 2004; Linhorst & Lübke, 2022; Schalk & Lavie, 2014).

**FIGURE 1:**
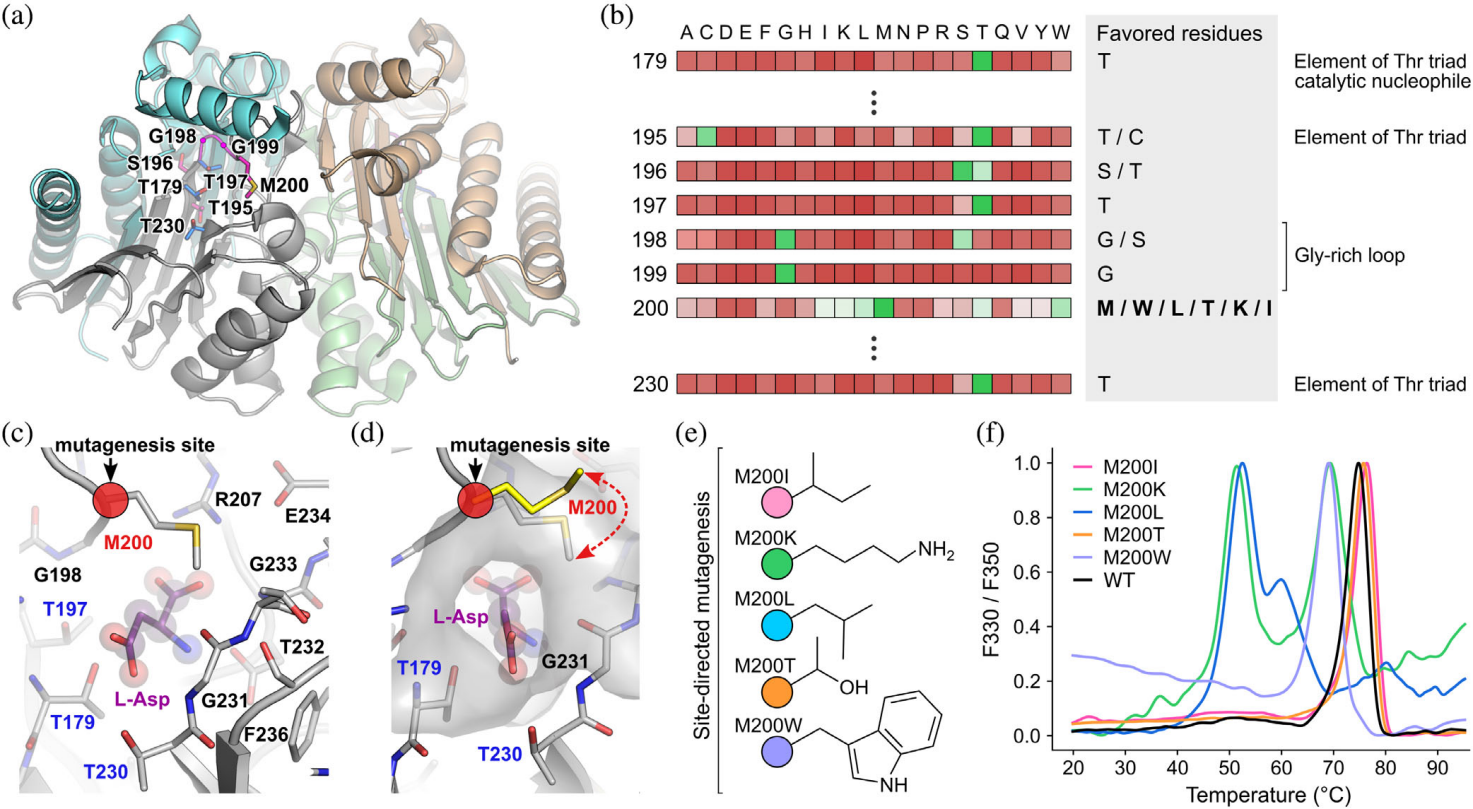
Site‐specific amino acid preferences near the active site of Class 2 L‐asparaginase. (a) Overall fold of mature WT EcAIII (PDB: 2zal): subunits α (chain A: light cyan; chain C: light orange), subunits β (chain B: gray; chain D: light green). (b) Position‐specific score matrix (PSSM) calculated based on Class 2 L‐asparaginase orthologs with reference to EcAIII numbering, showing how often a given residue is found at a specific position. Favored residues (occurring more often than their expected frequency) are shown in green, and avoided residues (occurring less often than their expected frequency) are shown in red. The complete PSSM profile across all L‐asparaginase sites is shown in Table S1. (c, d) A view of the active site of the EcAIII PDB structure 2zal. (c) Residues surrounding the product L‐Asp molecule (violet, spheres) in the active site: blue labels indicate the Thr triad, and the red circle indicates the Met200 mutation site. (d) The molecular surface surrounding the product/substrate cavity. Met200 (gray) acts as a gating residue that shuts off the active site entrance when the substrate/product is bound; the conformation of Met200 is as observed in unliganded EcAIII (yellow, PDB: 7qy6). (e) Graphical representation of the mutation types analyzed in this study. (f) Thermal stability and melting profiles of the new EcAIII variants analyzed in this study.

Ntn‐hydrolases are expressed as inactive precursors and develop enzymatic activity upon autoproteolytic maturation (Michalska et al., 2008; Nomme et al., 2012; Su et al., 2013). The precursor proteins consist of chains α and β forming two structural domains connected by a flexible linker. The mature proteins are αβ heterodimers, with a β‐sandwich architecture (Figure 1), that form the (αβ)_2_ heterotetramers. Although some studies of the structure–function relationship in Class 2 L‐asparaginases are available (Ajewole et al., 2018; Michalska et al., 2008; Nomme et al., 2014), there are still many aspects of the maturation process and the catalytic mechanism that remain unresolved.

Recent studies suggest that the therapeutic potential of L‐asparaginases is not limited to leukemias and can be applied to other types of cancer (Jiang et al., 2021). There is thus high interest in the design of new therapeutic L‐asparaginases (Van Trimpont et al., 2022; Wang et al., 2022). As Class 1 enzymes have already been extensively studied (Muneer et al., 2020; Patel et al., 2022), our interest has focused on Class 2 L‐asparaginases. In this project, we wanted to probe the role of selected residues in the activity of the *Escherichia coli* Class 2 L‐asparaginase, EcAIII, and thus establish the basis for future genetic engineering aimed at the production of novel biotherapeutics that could find applications in cancer therapy.

Protein engineering, especially site‐directed mutagenesis, is a common approach to elucidate the role of particular residues in protein stability, oligomerization, ligand binding preferences, or enzymatic activity. There are many ways to design mutations, but the most frequent approaches are based on multiple sequence alignments (Lehmann & Wyss, 2001) or superposition of protein structures (Bonarek et al., 2020). Such strategies can be successful but, in some cases, the mutations might have a detrimental effect on protein stability. The application of bioinformatics methods, as well as the use of machine‐learning strategies (Dauparas et al., 2022; Lipsh‐Sokolik et al., 2023) in the protein design process, makes classical site‐directed mutagenesis more powerful and effective (Anon, 2021; Schwersensky et al., 2020; Sumbalova et al., 2018).

**FIGURE 2:**
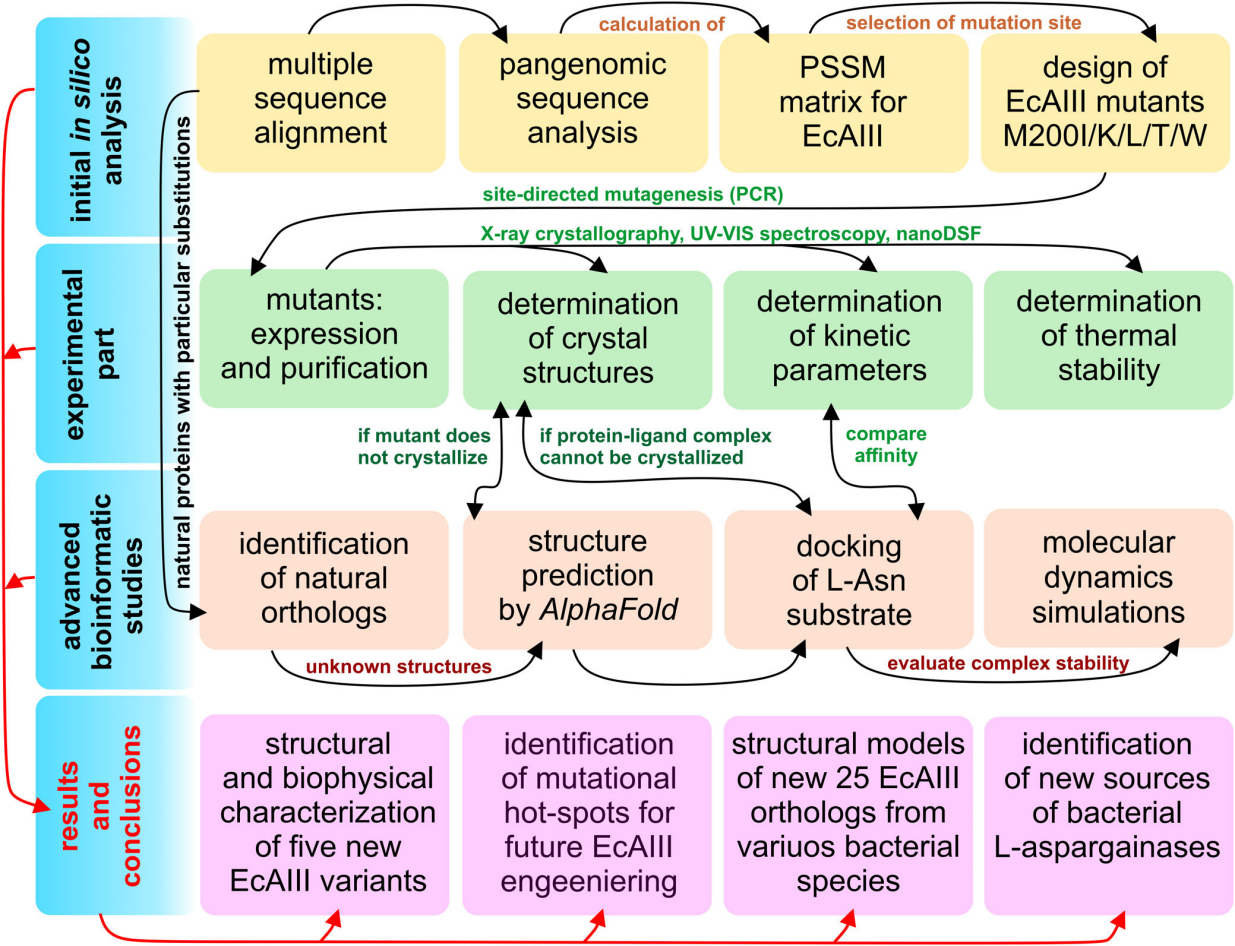
Schematic outline of the workflow used in this study. Different experimental and computational approaches were used to probe the effect of selected mutations on EcAIII properties. The experimental results complemented by *in silico* studies allow us to explain the properties of the new EcAIII variants and to understand how nature handles similar substitutions.

In this work, we show that pangenomic analyses, combined with molecular modeling and ligand docking, can facilitate the design of new and functional variants of EcAIII. For this purpose, we examined mutations that have been oversampled in the evolutionary history of more than 10,000 bacterial species (Table S1). We monitored sequence conservation in the close vicinity of the EcAIII catalytic threonine triad (Thr179‐Thr197‐Thr230), and selected the five most preferred substitutions. For these muteins, functional and structural studies were carried out. To complement the experimental results, molecular docking was used to clarify the mechanism of substrate binding. Additionally, we examined how other natural Class 2 L‐asparaginases adapt their active sites to the presence of residues that were selected for EcAIII mutagenesis in our experiment (Figure 2).

## 2 RESULTS

### 2.1 Sequence variability of Class 2 L‐asparaginases

Limiting the mutational search space to protein variants represented in the evolutionary history of the enzyme family is a proven strategy in protein engineering (Bershtein et al., 2008; Lehmann et al., 2000). Therefore, we used a recent collection of EcAIII orthologous sequences present in 11,435 bacterial species (Zielezinski et al., 2022) to generate a position‐specific scoring matrix (PSSM), which shows how often a given amino acid residue is found at a specific position across the orthologs (Table S1). Despite a broad phylogenetic distance separating the orthologs (Zielezinski et al., 2022), we observed over 40 highly conserved sites that are generally unlikely to be substituted, suggesting that these residues are critical for the structural and functional integrity of Class 2 L‐asparaginases.

In the close vicinity of the threonine triad and substrate binding site, we observed the highest variability across the orthologs at the position corresponding to Met200 in EcAIII (Figure 1b, Table S1). At this site, in more than half of the orthologs, methionine is replaced by one of the five residues: W, L, T, K, I, that occur more often than their expected frequency, indicating that these residues are specifically preferred in the evolutionary history of the enzyme. In the native EcAIII protein, Met200 is not directly involved in the catalytic process (Michalska et al., 2005, 2008) but rather acts as a “gate” to the active site and is involved in substrate stabilization (Figure 1d). Its conformation is affected by the presence of the substrate, therefore, the substitution of Met200 by other evolutionary favored residues might affect protein stability and substrate affinity.

### 2.2 Structural stability and enzymatic activity of the new EcAIII variants

All new EcAIII variants, that is, M200I, M200K, M200L, M200T, and M200W, retained the dimeric structure of the WT protein and the ability to undergo autoproteolytic maturation. However, a gel filtration (SEC) experiment revealed that mutant M200K tends to aggregate (Figure S1). The thermal stability determined by nanoDSF showed that mutants M200I and M200T had similar *T*_m_ to the WT protein, while variants M200W, M200L, and M200K (Figure 1f) had lower *T*_m_ (Table 1). Although the SEC and nanoDSF results suggested that mutant M200K had impaired folding, the L‐asparaginase activities clearly showed that all new EcAIII variants were able to hydrolyze L‐Asn. The determined *K*_M_ values were in the same (mM) range as for the WT protein (Table 1).

**TABLE 1:** *T*_m_ values and kinetic parameters determined for the new EcAIII variants.

Variant	*T*_m_ (^o^C)	*K*_M_ (mM)	*k*_cat_ (1/s)
EcAIII WT	74.4	22 ± 3	500.9 ± 22.3
M200I	75.9	35 ± 4	632.5 ± 37.9
M200K	51.4/69.2^a^	40 ± 9	127.1 ± 11.9
M200L	52.5	37 ± 5	194.7 ± 12.7
M200T	75.3	64 ± 6	217.3 ± 10.6
M200W	68.7	36 ± 10	290.1 ± 37.3

*Note*: The enzyme kinetic parameters are shown with ±SE determined from triplicate experiments.

^a^ *T*_m_ determined for the two thermal transitions shown in Figure 1f.

### 2.3 Crystal structures of the new EcAIII variants

We obtained crystals of four mutants M200I, M200L, M200T, and M200W. Despite many trials, we could not crystallize the M200K mutein. We determined one crystal structure for variant M200I and one for M200L, two crystal structures for the M200T mutant (orthorhombic M200T#o and monoclinic M200T#m), and two crystal structures of variant M200W (M200W#1 and M200W#2) representing the same symmetry (Table S2) but showing different conformation of residues near the mutation site.

**FIGURE 3:**
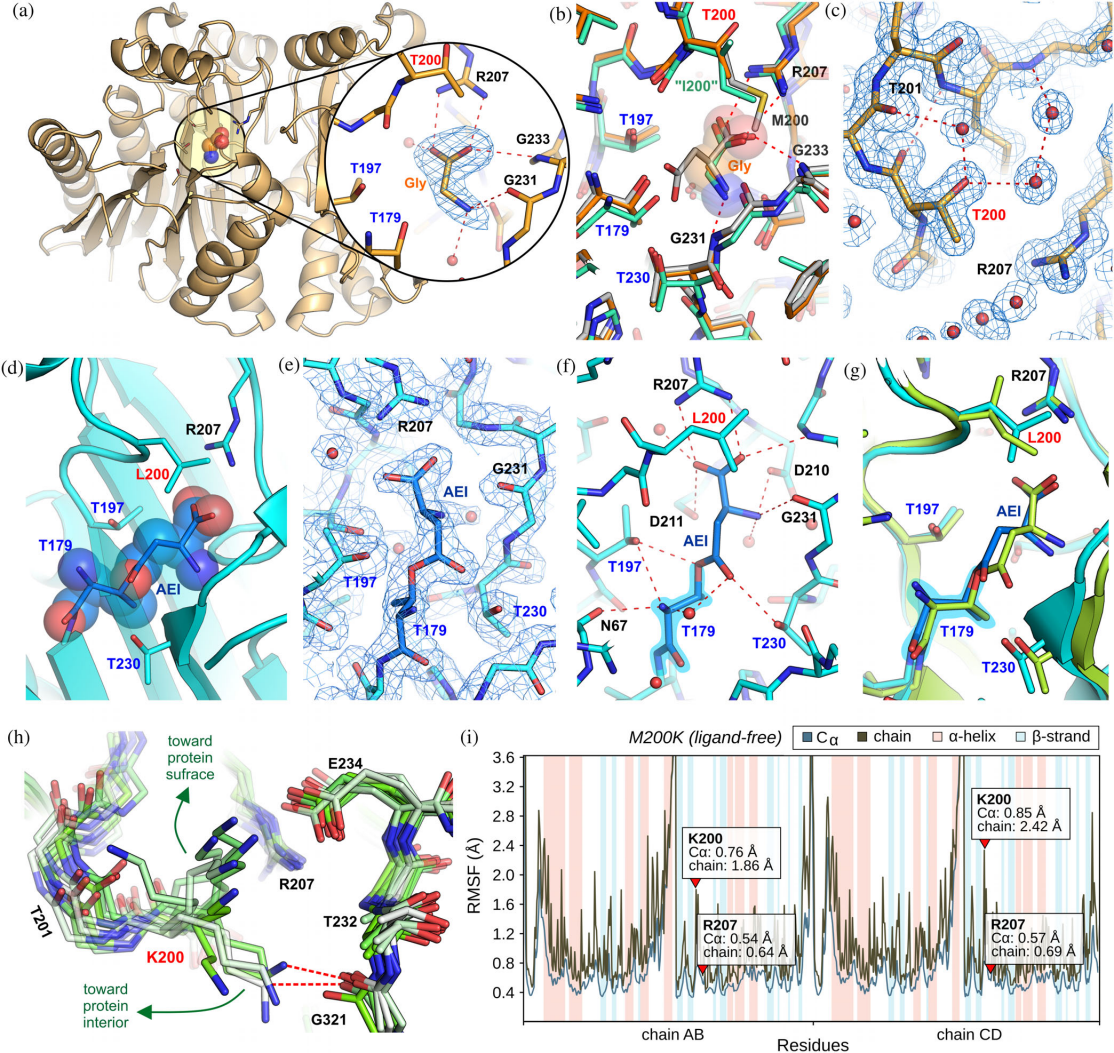
Crystal structures of mutants M200T, M200L and prediction of M200K structure. (a) 2Fo‐Fc electron density map (1.00 σ contour) near the Gly molecule identified in one of the active sites in the M200T#m structure (1.84 Å). (b) Superposition of the Gly complex M200T#m structure (orange), the Gly complex structure of the human protein HsAIII (green, PDB: 4osy), and WT EcAIII in complex with L‐Asp (gray, PDB: 2zal). (c) Quality of the 2Fo‐Fc electron density map (1.00σ contour) near the mutation site in the M200T#o structure (1.22 Å). (d) The acyl‐enzyme intermediate (AEI) in variant M200L. (e) 2Fo‐Fc electron density map contoured (at 1.00σ) around the substrate covalently bound to Thr179 (AEI). (f) H‐Bond network (red dashed lines) in the EcAIII active site with the covalently bound substrate (AEI). (g) Superposition of the acyl‐enzyme intermediates found in the crystal structures of the EcAIII mutant M200L (blue) and of the human protein HsAIII (green, PDB: 4o0h). (h) Predicted structure of the EcAIII variant M200K; possible conformations of the Lys200 side chain obtained after clustering the MD results; some conformers might form H‐bonds with the carbonyl oxygen of Gly231. (i) Diagram representing positional fluctuations of all amino acids for the M200K variant based on the MD simulations; RMSF is the root fluctuation calculated for the side chain of a given residue (chain) or only from the trace Cɑ.

In variants M200I and M200L, the new aliphatic side chain at position 200 was adjusted very well to the shape of the active site (Figure S2). Interestingly, in the structure of variant M200L in chain D, we identified an acyl‐enzyme intermediate (Figure 3). Substitution of Met200 by Thr also was neutral for the protein structure, however, it resulted in the formation of additional H‐bonds (Figure 3). In the second structure of this mutant, M200T#m, a glycine molecule was identified in two out of the four active sites in the asymmetric unit (Figure 3).

**FIGURE 4:**
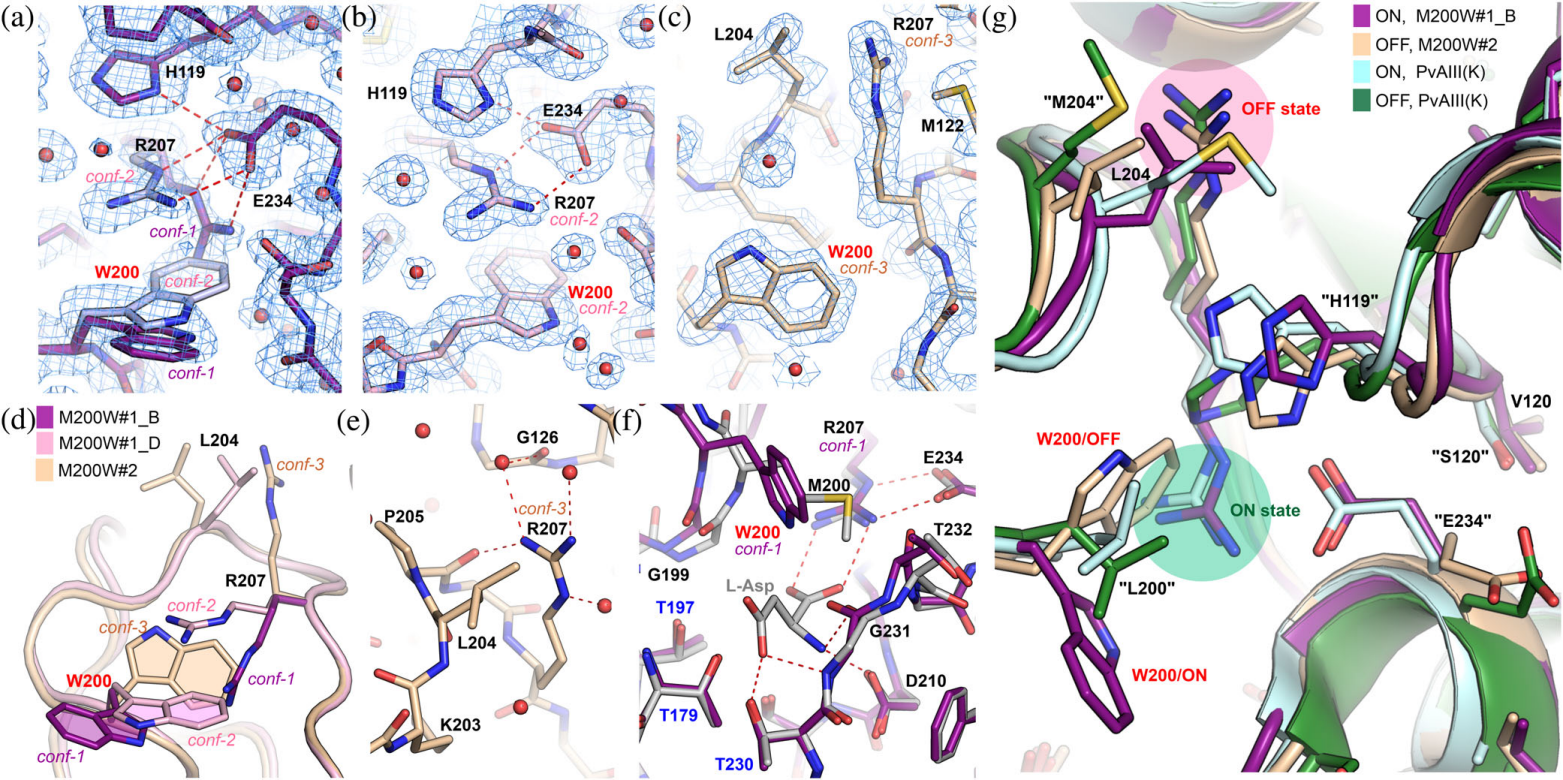
Crystal structures of mutant M200W. Conformational changes in the M200W structure (a) M200W#1, chain B; conformational disorder is visible for Trp200 and Arg207; conformers are marked by violet (conf‐1) and light violet (conf‐2); (b) M200W#1 (chain D, light pink), and structure (c) M200W#2 (chain B, beige). (d) Superposition of the structures M200W#1 and M200W#2 (as in panels a‐c), showing the different positions and conformations of Trp200 and Arg207. (e) H‐Bonds stabilizing the unusual conformation of Arg207 in structure M200W#2. (f) Superposition of structure M200W#1 (chain B) and EcAIII in complex with L‐Asp (PDB: 2zal); (g) The ON/OFF switch of K‐dependent plant L‐asparaginases; superposition of the M200W structures with Arg207 in the ON (violet) and OFF state (beige), together with the PvAIII(K) structures in the ON (light cyan, PDB: 4pu6) and OFF state (dark green, PDB: 4pv3).

The most important structural changes were found for mutant M200W. The crystal structures showed that Trp200 most significantly affected the position of Arg207 (Figure 4). In the native enzyme, Arg207 is crucial for substrate binding. We observed two types of the positional change of Arg207 (Figure 4d). In structure M200W#1 (in chain B), a positional disorder of Trp200 was observed, manifested as two possible conformations of the Trp200/Arg207 pair. When the indole ring of Trp200 was directed toward the protein surface (*conf‐1*, Figure 4d), the side chain of Arg207 took the position ready for substrate binding (Figure 4f). When the side chain of Trp200 was directed toward the protein interior, the side chain of Arg207 (*conf‐2*, Figure 4d) pointed toward the center of the active site, but its guanidinium moiety was shifted by ~3.2 Å toward the protein surface. This structural change was forced by the indole ring of Trp200, which squeezes in the space between Gly199 and Thr232 that is usually occupied by the natural Met200 side chain. In the M200W#2 structure, a more drastic structural rearrangement was observed, whereby the indole ring of Trp200 approached Lys203, pushing the side chain of Arg207 away (*conf‐3*, Figure 4d), into a groove between Met122 and Leu204 (Figure 4c). This conformational modification also affected the conformation of Glu234, which rotated by ~180° toward Arg238.

### 2.4 AI prediction of the structure of variant M200K of EcAIII

Due to the propensity of the M200K variant to aggregate, we predicted its structure with AlphaFold2 (Jumper et al., 2021). The results show that the overall structure of the M200K variant is the same as the WT protein (rmsd for Cɑ 0.23 Å). Analysis of the molecular interactions near the mutation site revealed that Lys200 could be stabilized by an H‐bond to the carbonyl oxygen of Gly231. However, molecular dynamics simulations show that the side chain of Lys200 is labile and fluctuates, occupying different positions (Figure 3). The wobbliness of Lys200 can be attributed to electrostatic repulsion by the positively charged Arg207. Although in the EcAIII structure, Glu234 is present in the close proximity of Lys200, the computational model shows that the distance between these two residues is too large for a productive H‐bond (Figure 3).

### 2.5 Validation of the molecular docking method

**FIGURE 5:**
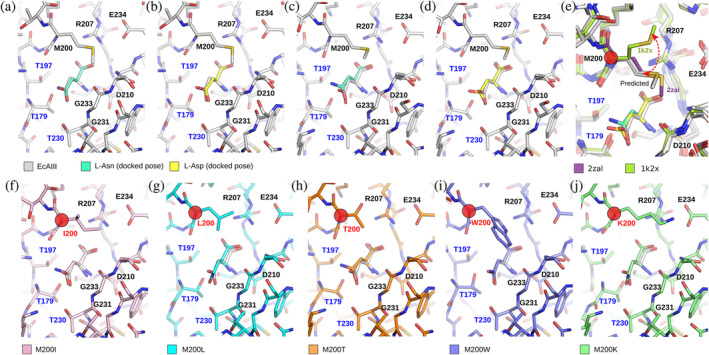
Validation of the molecular docking method and docking studies of ECA III variants. (a–d) Validation of molecular docking method. The best docking results were obtained for molecular docking of L‐Asn (a) and L‐Asp (b) using SP docking mode compared with the reference orientation of L‐Asp in the PDB EcAIII structure 2zal. (c, d) The best docking results of (c) L‐Asn and (d) L‐Asp were obtained from IFD protocol with reference orientation of ligand L‐Asp. (e) Prediction of conformational changes upon ligand binding in Met200 side chain obtained during IFD docking, compared with experimental orientation of Met200 residue from ECAIII/L‐Asp (2zal) and ligand‐free ECAIII (1k2x) (f–j) Molecular docking of L‐Asn to EcAIII variants obtained using the IFD protocol: Best obtained L‐Asn poses for (f) M200I, (g) M200L, (h) M200T, (i) M200W, and (j) M200K variant with the reference orientation L‐Asp ligand. Red circles mark residue corresponding to Met200 in the EcAIII molecule. In all panels, reference ligand orientation is shown as gray sticks.

Prior to molecular docking simulations, we tested the algorithm using a crystal structure of the ligand‐free EcAIII protein (PDB: 1k2x) and its complex with L‐Asp (PDB: 2zal). Specifically, we performed docking simulations for L‐Asn and L‐Asp to a designated binding site using two different algorithms: the SP docking mode and the IFD protocol. The SP docking mode was used for the 2zal structure as input, while the IFD protocol was applied to the structure without L‐Asp (PDB: 1k2x), mainly to verify the prediction of conformational changes induced by ligand binding. The results show comparable docking structures of the two ligands (Figure 5a–d), with the highest‐scoring pose (Table S5) reproducing the experimental orientation of L‐Asp in the PDB structure 2zal. The results obtained using the IFD protocol also correctly predicted the conformational changes within the ligand binding site, in particular the position of Met200 (Figure 5e). The rmsd value for the Met200 position was 0.86 Å for L‐Asn and L‐Asp, with respect to the orientation in the experimental structure 2zal (calculated for the methionine side chain non‐H atoms only).

### 2.6 Docking calculations for the new EcAIII variants

Despite numerous co‐crystallization trials, we were unable to obtain crystals of the EcAIII mutants in a complex with the L‐Asn substrate or L‐Asp product (except for the acyl‐enzyme intermediate in M200I). Therefore, we used the IFD protocol to dock the substrate into the binding site of the crystal structures of the EcAIII mutants and to the predicted model of the M200K mutant. The results show very similar docking scores and binding energies for all variants (Table 2) with only minor atomic shifts (Figure 5).

**TABLE 2:** Docking score, binding energy, and rmsd values determined for molecular docking of the L‐Asn substrate to the EcAIII variants studied in this work.

Variant	Docking score^a^	*E*_Bind_ (kcal/mol)^b^	rmsd (Å)^c^
EcAIII WT	−7.22	−28.57	0.54
M200I	−7.29	−33.37	0.45
M200K	−6.76^d^	−30.20	0.73
M200L	−6.96	−29.50	0.64
M200T	−6.75^d^	−28.54	0.61
M200W	−7.39	−30.69	0.30

^a^ Docking score obtained from IFD molecular docking protocol.

^b^ MM‐GBSA binding energy estimation evaluated from Prime module.

^c^ rmsd for the L‐Asn docking pose calculated over non‐H atoms of the L‐Asp ligand in the PDB 2zal structure.

^d^ Based on MD data, the ligand pose is unstable and ligand diffuses from the binding site at the end of MD simulation (Table S5).

Superposition of the docking results with the crystal structure of the EcAIII/L‐Asp complex (PDB: 2zal) revealed that in variants M200I and M200T, the position of the docked substrate is almost identical to the position of the product (L‐Asp in the crystal structure; Figure 5f,h). In variant M200L, the ɑ‐carboxylate group of the substrate was shifted by about 0.53 Å with respect to the position in the crystal structure (Figure 5g). A positional shift (0.8 Å) of the substrate ɑ‐carboxylate group was also predicted in mutant M200K, although in this case, an H‐bond between Lys200 and Glu234 reduced the movement of the substrate (Figure 5j).

In the M200W variant, with a bulky Trp200 residue, the active site was flexible enough to accommodate the substrate. The L‐Asn molecule was anchored by H‐bonds to the guanidinium group of Arg207. Since the crystal structure M200W#2 revealed that the Trp200 side chain can also push Arg207 away from the active site (Figure 4), we docked the substrate to the active site of structure M200W#2. This experiment showed that the substrate can be held in place by H‐bonds to Thr179, Asp210, Thr230, and Gly231, but a molecular dynamics (MD) simulation revealed that the ligand would quickly dissociate from the active site (Figure S3). It appears that the presence of the side chain of Arg207 is necessary to form a strong salt bridge with the ɑ‐carboxylate group of the substrate to ensure effective catalysis.

### 2.7 Exploring the structural space of EcAIII orthologs

We examined how EcAIII orthologs have adapted their active sites during evolution to the presence of different residues at position “200.”* Orthologous sequences were identified among 15,600 of bacterial genomes (Zielezinski et al., 2022) and their structures were predicted by AlphaFold2 (Jumper et al., 2021). For each of the five residues, K, I, L, T, W at position “200,” we selected for modeling five orthologs. The selection was based on two criteria: (i) distinct combinations of amino acid substitutions at 17 positions within the binding site of the substrate, and (ii) the overall sequence identity between 25% and 65% to the reference EcAIII sequence (Table S3). Currently, there are a limited number of Class 2 L‐asparaginases structures available (da Silva et al., 2018; Sajed et al., 2022; Sharma et al., 2022), so we have assembled a library of structures of predicted models and provided them as Supplementary Material.

In general, all predicted proteins had a fold similar to EcAIII reference, and retained the positions of “Arg207” and “Glu234,” as well as the conformation of the sodium‐binding loop (Figure S4). However, the type of residue at position “200” often affected the chemical character of residue “232” in subunit β, and “119” in subunit ɑ. In the analyzed proteins possessing “Thr200,” the changes were found at position “232,” where Asp and Gln occur interchangeably, and at position “211” where Ala, Val or Thr were found (Table S3). In orthologs with “Leu200,” “Thr232” was replaced by Asp, Tyr or His. Analysis of the predicted structures with “Ile200” show that “Thr232” can be replaced by Ala, Ser, or Met. These substitutions seem to be neutral for substrate binding. In some orthologs, we detected changes in the threonine triad, e.g. in the protein from *C. humilis* “Met179” was found instead of “Thr179,” in the *Vibrio* protein “Thr197” was replaced by Ala, while in the *Opitutus* ortholog—by Ser (Table S3).

**FIGURE 6:**
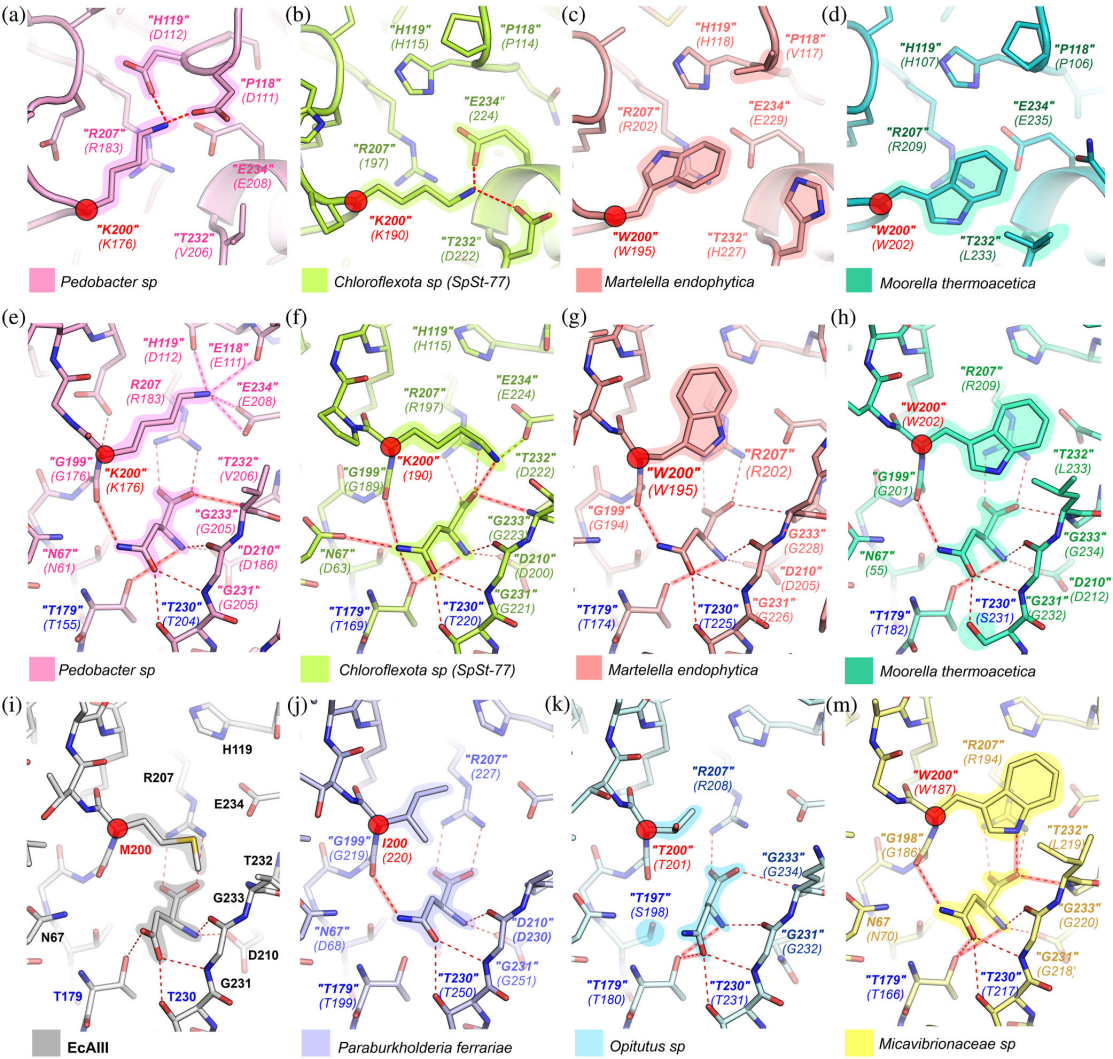
Structure prediction for selected orthologs and docking of substrate to selected orthologs. H‐Bonds between “Lys200” and neighboring residues in protein form (a) *Pedobacter* and (b) *Chloroflexota* sp. (SpSt‐77). Orientation of residues in the close vicinity of “Trp200” in protein from (c) *M. endophytica* and (d) *M. thermoacetica*. Docking of substrate (L‐Asn) to protein from (e) *Pedobacter* possessing “Lys200”; (f) *Chloroflexota* sp. (SpSt‐77) possessing “Lys200”; (g) *M. endophytica* possessing “Trp200”; (h) *M. thermoacetica* possessing “Trp200.” (i) Complex of WT EcAIII and product L‐Asp (PDB: 2zal). Docking of the substrate to protein (j) *P. ferrariae* possessing “Ile200”; (k) *Opitutus* possessing “Thr200”; (m) *Micavibrionaceae* sp possessing “Trp200.” H‐bonds are marked by red dashed line; red‐outlined H‐bonds in the structures of orthologs are different than those observed in WT EcAIII (PDB: 2zal, panel i); in panel e and f green and pink outlined H‐bonds are between “Lys200” and other residues in the ortholog. Residue corresponding to the EcAIII sequence is marked in bold italics in quotation marks, while italics in parentheses mark the positions of residues in the selected ortholog sequences. Red circles mark residue corresponding to Met200 in the EcAIII molecule.

The most significant changes were found in natural proteins carrying “Lys200” and “Trp200” (Figure 6). In these orthologs, the residues of the threonine triad were retained, with the exception of the *M. thermoacetica* protein (Table S3). To facilitate the accommodation of “Trp200,” an aliphatic “Leu232” or polar “His232” was found instead of “Thr232.” In the predicted structures, two different conformations of “Trp200” were observed (Figure S4): with the “Trp200” side chain positioned between “His232” and “His119” (*M. endophytica*), or with the position of the indole ring almost perpendicular to the guanidinium group of “Arg207” (*M. thermoacetica*). In the orthologs with the “M200K” substitution, the most important changes were observed in the region of “Thr232” and “Ser117‐Met121.” It seems that the presence of a positively charged “Lys200” is compensated by the insertion of negatively charged residues at H‐bond distances. Three types of such substitutions were observed: “T232D,” “P118E,” and “H119D” (Figure 6a,b).

### 2.8 Docking studies of selected orthologs of EcAIII

To investigate how natural proteins accommodate the substrate, we performed docking experiments with the predicted orthologous structures using the same approach as for the EcAIII variants. L‐Asn could be docked to all orthologs with a binding pose similar to the reference structure (PDB: 2zal) but the MD simulations indicated that in proteins from *C. humilis*, *Pedobacter* sp., *Phenylobacterium* sp., *Martelella endophytica*, *Micavibrionaceae* sp., *S. stellata*, and *Martelella limonii* the substrate was unstable in the active site and diffused away from it during the simulation (Table 3, Table S5).

**TABLE 3:** Docking score, binding energy, and rmsd values determined for molecular docking of the L‐Asn substrate to EcAIII orthologs.

EcAIII orthologs/species^a^	Working name of bacteria^b^	Docking score^c^	*E*_Bind_ (kcal/mol)^d^	rmsd (Å)^e^
“M200I”				
*Izhakiella capsodis*	*Izhakiella capsodis*	−6.82	−30.53	0.79
*Bordetella B ansorpii A*	*Bordetella ansorpii*	−6.67	−34.31	0.66
*Paraburkholderia ferrariae*	*Paraburkholderia ferrariae*	−7.08	−31.23	0.78
*Caballeronia humilis*	*Caballeronia humilis*	−7.21^f^	−33.20	0.82
*Vibrio sp006124995*	*Vibrio* sp. *unclassified*	−6.76	−22.85	0.61
“M200L”				
*Gynuella sunshinyii*	*Gynuella sunshinyii*	−7.36	−34.08	0.99
*Larkinella sp004366505*	*Larkinella* sp. *unclassified*	−7.10	−29.94	0.55
*BM003 sp002868855*	*Chromatiales* sp. *unclassified*	−7.19	−30.84	0.53
*YR4‐1 sp011059145*	*Halalkalibacterium roseum*	−6.87	−27.67	0.50
*Schlesneria sp903904835*	*Schlesneria* sp. *unclassified*	−7.06	−32.05	0.89
“M200T”				
*UBA2146 sp002311975*	*Candidatus Marinimicrobia*	−6.84	−27.33	0.35
*Udaeobacter sp003219395*	*Udaeobacter* sp. *unclassified*	−7.18	−30.89	0.66
*Phenylobacterium sp013822795*	*Phenylobacterium* sp. *unclassified*	−6.33^f^	−29.32	0.73
*Opitutus sp903877135*	*Opitutus* sp. *unclassified*	−6.21	−26.18	1.13
*Niveispirillum irakense*	*Niveispirillum irakense*	−6.90	−29.41	0.40
“M200K”				
*SpSt‐77 sp011362935*	*Chloroflexota* sp. *unclassified*	−9.04	−37.51	1.13
*Mucilaginibacter gossypiicola*	*Mucilaginibacter gossypiicola*	−6.92	−30.44	1.07
*Pedobacter sp009765875*	*Pedobacter* sp. *unclassified*	−7.11^f^	−30.63	0.86
*Microcystis aeruginosa_E*	*Microcystis aeruginosa*	−6.80	−31.54	0.81
*Myroides guanonis*	*Myroides guanonis*	−7.46	−31.17	0.93
“M200W”				
*Martelella endophytica*	*Martelella endophytica*	−7.39^f^	−36.92	0.57
*Moorella thermoacetica_A*	*Moorella thermoacetica*	−7.29	−33.73	0.63
*Sagittula stellata*	*Sagittula stellata*	−6.46^f^	−27.61	0.46
*UM‐FILTER‐47‐13 sp002789675*	*Micavibrionaceae* sp. *unclassified*	−7.04^f^	−30.38	0.80
*Martelella limonii*	*Martelella limonii*	−6.87^f^	−34.62	0.64

^a^ Species names from the Genome Taxonomy Database.

^b^ As many presented bacteria are unclassified yet, we use simplified working names, for example, *Vibrio* sp006124995 is named *Vibrio* sp. *unclassified (Vibrio species unclassified)*.

^c^ Docking score obtained from IFD molecular docking protocol.

^d^ MM‐GBSA binding energy estimation evaluated from the Prime module.

^e^ rmsd for the L‐Asn docking pose calculated over non‐H atoms of the L‐Asp ligand in the PDB 2zal structure.

^f^ Based on MD data, the ligand pose is unstable and ligand diffuses from the initial binding site (Table S5).

We observed that “Ile200” in orthologs can assume different conformations that may affect the orientation of the substrate amide group (Figure 6). The position of the substrate in the orthologs with “Leu200” is almost identical to the position of L‐Asp in the crystallographic PDB model 2zal. This reflects the relative rigidity of “Leu200,” which occupies the same space as Met200 in EcAIII reference. In the orthologs with “Thr200,” different orientations of the Thr side chain were observed, although they did not affect the position of the substrate molecule.

In the orthologs with “Lys200,” we found variable conformations of the lysine side chain. In the protein from *Chloroflexota* sp. (SpSt‐77), the ɑ‐carboxylate group of the substrate was H‐bonded not only to “Arg207,” but also to “Lys200” (Figure 6). Such configuration of H‐bonds of H‐bonds resulted in a rotation of the L‐Asn ɑ‐carboxylate group and its shift by ~1.30 Å toward “Thr232” with respect to the 2zal structure. This movement of the ɑ‐carboxylate group caused a shift of the L‐Asn amide and reorganization of the entire system of H‐bonds involved in substrate stabilization (Figure 6). We observed similar changes in the protein from *Pedobacter* sp.; however in this case, “Lys200” was not H‐bonded to the substrate but to neighboring Glu and Asp residues (Figure 6). These differences in the interactions of “Lys200” with the substrate are the consequence of the initial position of “Lys200” in the unliganded orthologs (Figure 6a,b).

The orthologs with “Trp200” showed a positional shift of the substrate in proteins from *M. endophytica* and *M. thermoacetica* (Figure 6). In these structures, the side chain of “Trp200” pushed the L‐Asn molecule toward the nucleophilic “Thr179” at a distance allowing H‐bond formation between the Oɣ of “Thr179” and N atom of the substrate (Figure 6). In the protein form *Micavibrionaceae* sp., an H‐bond between “Trp200” and the substrate was present (Figure 6m).

## 3 DISCUSSION

### 3.1 Nature‐inspired mutagenesis enabled the design of functional EcAIII variants

The EcAIII enzyme from *E. coli* is the prototypic Class 2 Ntn‐amidohydrolase. We focused our studies on the role of Met200, which appears to be a substrate‐stabilizing residue in the EcAIII molecule (Figure 1). We produced a series of EcAIII variants with Met200 replaced by five residues found most frequently at this position in bacterial orthologs of EcAIII. The mutations were selected on the basis of PSSM analysis, which showed that Lys, Ile, Leu, Thr, and Trp are preferred in nature at position “200.” We assumed that these substitutions may have a beneficial effect on protein activity, as natural selection favors stable and active proteins.

To become enzymatically active, Ntn‐hydrolases must undergo autocatalytic maturation, but the initiation of this cleavage process is complex (Loch et al., 2022; Michalska et al., 2008). Our experiments showed that all new EcAIII variants were able to fully maturate to subunits α and β, so the type of residue at position 200 does not influence the EcAIII maturation process. More important for auto‐maturation are residues located at positions 210 and 211 in the EcAIII molecule, as they are placed deeper in the active site and closer to the nucleophilic Thr179 (Loch et al., 2022).

On the other hand, the type of residue at position 200 affected the thermal stability, especially of EcAIII variants M200K, M200L, and M200W (Table 1). The thermal destabilization of mutant M200W can be attributed to the presence of a bulky indole ring almost in the center of the active site, which disturbs the natural H‐bond pattern in this region. The predicted structure of variant M200K showed that Lys200 is highly disordered (Figure 3h). These fluctuations are the result of electrostatic repulsions between Lys200 and Arg207 (or His119) and might be responsible for the low *T*_m_ value. The structure of variant M200L showed that Leu200 is located very close to Arg207, which might lead to unfavorable interactions between these two side chains. Mutant M200I has slightly increased *T*_m_, which might be explained by the favorable position of Ile200 relative to Arg207. The increased thermal stability of variant M200T can be attributed to the favorable H‐bond pattern formed in the presence of Thr200 (Figure 3c). As adequate thermal stability is essential for enzymes with potential medical applications (Tandon et al., 2021), the EcAIII variants M200I and M200T appear to be promising candidates for further engineering aimed at increasing half‐life of the enzyme in the physiological fluids.

### 3.2 EcAIII substrate binding is not significantly affected by substitutions at position 200

The mechanism of L‐Asn hydrolysis is common to all Class 2 L‐asparaginases (Borek et al., 2004; Li et al., 2012; Michalska et al., 2005, 2006; Nomme et al., 2014; Schalk & Lavie, 2014). Our results (Table 1) confirmed that all new EcAIII variants were active, with *k*_cat_ of the same order as for WT EcAIII; thus, the substitutions of Met200 did not significantly affect the kinetics of L‐Asn hydrolysis. It is also apparent that single substitutions at position 200 are not sufficient to increase the substrate affinity of EcAIII.

As we were unable to crystallize complexes between the EcAIII variants and L‐Asn/L‐Asp (with the exception of M200L, Figure 3d), we used molecular docking to analyze the mode of substrate binding in the active site. The results confirm that all new EcAIII variants can accommodate the substrate (Figure 5). Analysis of the predicted protein‐substrate complexes confirmed that residue 200 is not crucial for substrate binding; however, small conformational and positional shifts of the substrate molecule were observed in the presence of the mutations (Figure 5). The MD data revealed that the L‐Asn pose in the M200K and M200T mutants is unstable (Table 2; Table S5), and that the substrate diffuses away from the active site at the end of the MD simulation. However, our *in vitro* experiments did not show any unusual behavior of the M200K and M200T mutants in the kinetic assays, although in SEC filtration the M200K variant had a tendency for aggregation.

The docking results revealed that binding of L‐Asn was even possible in variant M200W, although the substrate was stabilized in the active site only when Arg207 was directed toward the active site interior (enzyme in ON‐state, see Section 3.3) (Figure 4). The most important for proper substrate orientation is Arg207, as previously noted (Loch et al., 2022). The presence of Lys or Trp at position “200” might additionally stabilize the substrate, but this effect was observed only in natural orthologs possessing other favorable mutations in the close vicinity (Figure 6). These findings indicate that the EcAIII variants M200W and M200K could be interesting candidates for future engineering aimed at increasing the number of interactions with the substrate that could potentially affect the *K*_M_ value. However, M200W and M200K mutants should carry additional substitutions to allow the correct positioning of Lys200 and Trp200 close to the active site.

The crystal structure of variant M200L allowed us to observe the β‐acyl‐enzyme intermediate. This provides the first experimental evidence that in EcAIII, analogously to human L‐asparaginase HsAIII (Nomme et al., 2014), L‐Asn hydrolysis is a two‐step reaction, with the formation of an intermediate product. A comparison of the acyl‐enzyme intermediate in the M200L crystal and in a similar crystal of HsAIII (Nomme et al., 2014) (Figure 3g), shows that the structures are almost identical, despite the relatively low level of sequence identity (37.5%). In contrast to their bacterial homologs, mammalian Class 2 L‐asparaginases (such as HsAIII), when expressed in a bacterial host, do not undergo spontaneous maturation. Their autocleavage can be induced by incubation with glycine (Li et al., 2016; Su et al., 2013). Although all the EcAIII variants underwent processing in the expression host, we observed that the addition of glycine noticeably facilitated the crystallization of the M200T mutant.

In the structure M200T#m, glycine molecules were found in two out of four active sites. The glycine position was almost identical to that observed in the HsAIII enzyme (Figure 3b). This position of the Gly molecule overlaps with the ɑ‐carboxylate group of the substrate/product (Figure 3b). These findings indicate that glycine could act as a competitive inhibitor of Class 2 L‐asparaginases. Notably, the presence of glycine can impact the efficacy of therapeutic (Class 1) L‐asparaginases and antagonize their therapeutic value (Chattopadhyay et al., 1973; Ryan & Sornson, 1970). This important issue has been overlooked so far, as L‐asparaginases are typically engineered to eliminate their detrimental glutaminase side activity (Ln et al., 2011; Nguyen et al., 2016). Therefore, future engineering of L‐asparaginases to reduce their glycine affinity may be beneficial in enhancing their therapeutic efficacy.

### 3.3 Trp200 in EcAIII might induce a conformational “switch” as in K‐dependent enzymes

Class 2 of L‐asparaginases includes K‐dependent and K‐independent enzymes (Loch & Jaskolski, 2021). The K‐dependent enzymes can be found in some plants and show maximum catalytic efficiency at high potassium concentrations (Ajewole et al., 2018). Sensitivity to K^+^ is mediated through the presence of the Activation Loop (AL), which has a specific sequence that allows for the binding of K^+^ (Bejger et al., 2014). The AL is absent in EcAIII. Coordination of K^+^ activates a “catalytic switch” that stabilizes “Arg207” in a position optimal for substrate binding (ON‐state) (Figure 4g). The “catalytic switch” is comprised of three residues, “His119,” “Arg238,” and “Glu234” (Figure 4g). In the absence of K^+^, K‐dependent enzymes are in the OFF‐state and their catalytic efficiency is reduced (Ajewole et al., 2018).

When the K‐dependent enzymes are in the OFF‐state, “Arg207” is rotated away from the active site and takes a position almost identical to that found in structure M200W#2 (Figure 4g). This observation indicates that the conformation of “Arg207” in the OFF‐state may also be achieved in K‐independent enzymes (Figure 4). As the M200W variant retained the ability to hydrolyze L‐Asn, it may be concluded that in a predominant population of the EcAIII molecules in solution, Arg207 is in a position compatible with the ON‐state (structure M200W#1, Figure 4). The OFF‐state conformation in M200W#2 is not a crystallization artifact, as structures M200W#1 and M200W#2 have the same space group and were obtained at similar conditions. The EcAIII molecule in the OFF‐state is not able to tightly bind the substrate molecule, which was confirmed by our docking results for crystal structure M200W#2 (see Section 2.6).

### 3.4 Natural adaptations and sequence variability in the active site in EcAIII orthologs

Natural adaptations of protein sequences to environmental conditions are the result of multiple substitutions appearing in the course of evolution (Colautti & Lau, 2015; Gregory & Ryan Gregory, 2009; Lenski, 2017). Therefore, in this work, we investigated the sequence variability in selected EcAIII orthologs. The analyzed sequences were selected according to their similarity to EcAIII. Although the taxonomic affiliation of some of the species listed in Table 3 and S6 remains unclear, as they have been discovered only recently, the bacteria carrying EcAIII orthologs with Lys, Leu, Ile, Thr, or Trp residues at position “200” can be found in highly diverse habitats (Table S6).

On the other hand, the crystal structures of mammalian and plant enzymes deposited in the PDB show that the “Met200” in mammalian proteins might be replaced with “Leu200” in the human (HsAIII) (Nomme et al., 2012) and guinea pig (CpAIII) (Schalk & Lavie, 2014) protein, while in common bean (PvAIII[K]) (Bejger et al., 2014) and yellow lupin (LlAIII) (Michalska et al., 2006) enzymes “Ile200” is present. These substitutions are the same as those analyzed in our work, and indicate that the mammalian and plant enzymes may have a similar distribution of residue types in the conserved areas as detected in bacterial proteins. Additionally, the data in Table S6 indicate that bacteria living in halophilic and thermophilic environments as well as in the rhizosphere could serve as valuable sources of L‐asparaginases with interesting properties, as has been recently shown for *Rhizobium etli* L‐asparaginase (Loch et al., 2021; Moreno‐Enriquez et al., 2012).

Our molecular modeling showed that EcAIII orthologs with “Leu200,” “Ile200,” or “Thr200” did not show any important changes near the active site. On the other hand, the predicted structures of orthologs with “Lys200” showed that the presence of the positively charged Lys is compensated in nature by the addition of negatively charged residues, which form H‐bonds or salt bridges that stabilize the lysine side chain (Figure 6). The absence of such negatively charged partners in the structure of EcAIII variant M200K explains its decreased *T*_m_ and the problems with its crystallization, which could be linked to local disorder detrimental to the formation of a crystal lattice. Furthermore, the modeled structures of orthologs with “Trp200” revealed that the presence of the bulky indole ring is not associated with a drastic rearrangement in the active site area. Substitutions that might be interpreted as adaptation to “Trp200” are only present at position “232”: His, Leu, or Ile instead of “Thr232.” These side chains have more conformational freedom than the native Thr, and might, therefore, support the proper positioning of “Trp200” when the substrate is bound.

Moreover, some of the EcAIII orthologs seem to lack an enzymatically competent active site, suggesting that they may have lost their L‐asparaginase function. In some proteins (Table S3; Table S6) one of the threonines from the threonine triad is replaced by Ser, demonstrating that the threonine triad can be replaced by a serine–threonine triad (Table S3) in configurations such as “Thr179”‐”Ser197”‐”Thr230” (as in the *Opitutus* sp. ortholog) or “Thr179”‐”Thr197”‐”Ser230” (as in *M. thermoacetica* ortholog). The enzymes with a serine–threonine triad appear to be functional, but it is difficult to predict their kinetic parameters without experimental verification. The function of the individual residues forming the threonine triad has previously been investigated by site‐directed mutagenesis (Li et al., 2012, 2016; Loch & Jaskolski, 2021; Michalska et al., 2008; Nomme et al., 2014; Su et al., 2013). However, only two studies examined the effect of replacing one of the threonines from the threonine triad with serine. These studies were focused on the human enzyme HsAIII (Nomme et al., 2014) and found that the substitution of “Thr179” by serine prevented the autocatalytic maturation of HsAIII. Substitution of “Thr197” by Ser resulted in the reduction of autoprocessing rate and L‐asparaginase activity. The same effect was observed in case of substitution of “Thr230” by Ser in HsAIII (Li et al., 2016).

### 3.5 *In silico* modeling of substrate binding to EcAIII orthologs

We tested if the EcAIII orthologs are able to accommodate the substrate in the active site. The docking tests performed for EcAIII (Section 2.6) showed that the IFD approach correctly reproduced the experimental ligand orientation and conformational (Figure 5). The simulations confirmed that L‐Asn can be bound to all orthologs in positions similar to those observed for L‐Asp in the PDB crystal structure 2zal (Table 3). The calculated substrate‐binding energy was very similar for the ortholog‐substrate complexes and EcAIII reference (Table 3), suggesting similar substrate affinity.

The most interesting results were obtained for the orthologs carrying the “M200W” and “M200K” substitutions. In some of these proteins, H‐bonds between “Lys200” or “Trp200” and the substrate were identified (Figure 6). This observation suggests that the substrate might be bound more efficiently in the presence of “Lys200” or “Trp200” (Figure 6). In these orthologs, we also found an additional H‐bond between the nucleophilic “Thr179” and the substrate. We note that such an interaction might affect the nucleophilic character of “Thr179.” Therefore, the substitutions “M200W” and “M200K” may be beneficial for substrate stabilization in the active site of Class 2 L‐asparaginases, but to achieve such a positive effect, additional mutations should be simultaneously present to prevent large‐amplitude fluctuations of “Lys200” or switching of the enzyme to the OFF‐state by “Trp200.”

### 3.6 Probing EcAIII active site by random and nature‐inspired site‐directed mutagenesis

**FIGURE 7:**
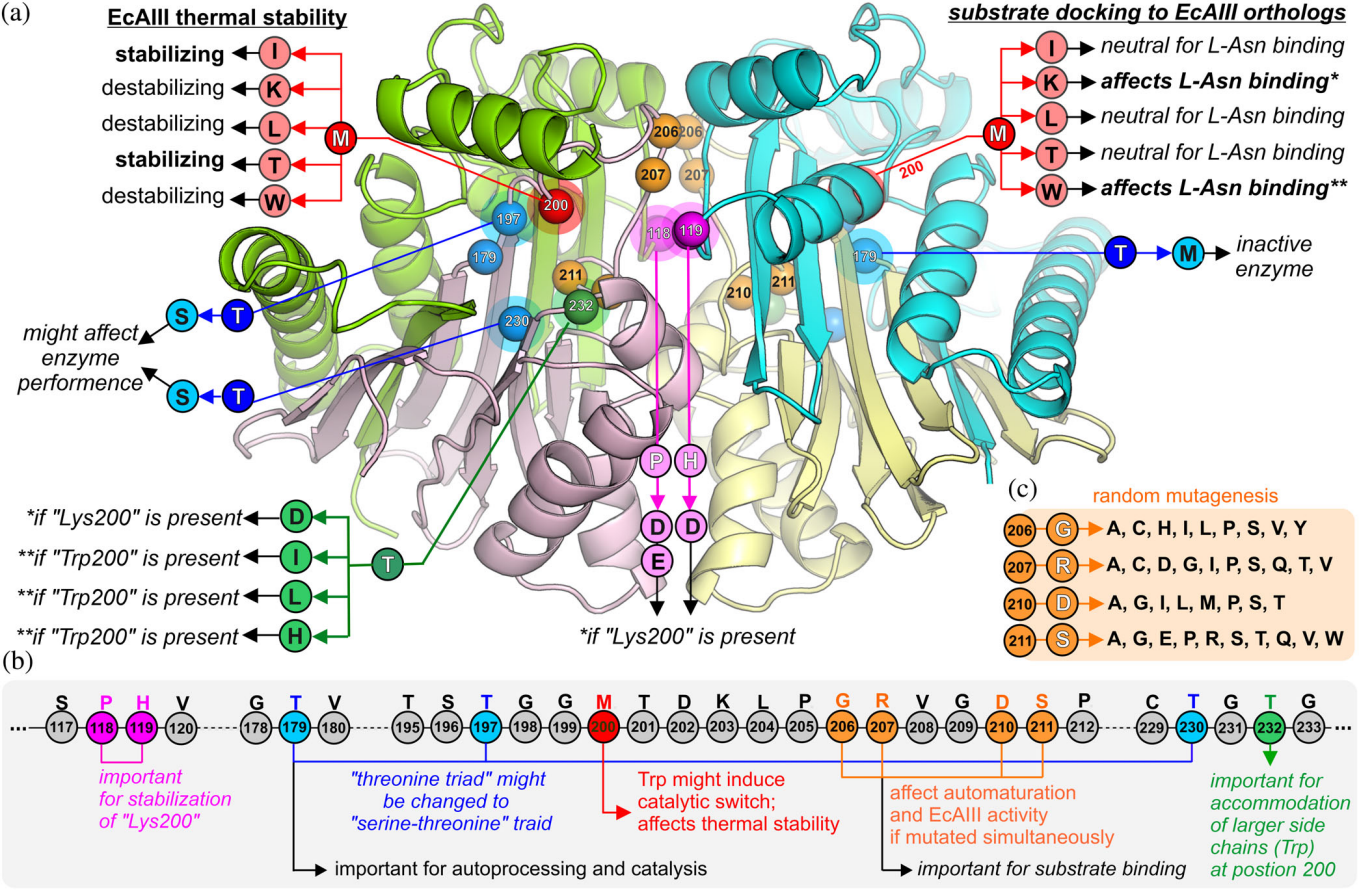
Probing the EcAIII active site by mutagenesis and engineering hot‐spots. The presented and analyzed mutations are the same (and have the same effects) in both subunits, but for clarity they have been marked in one (either) subunit of the dimer. (a) EcAIII structure with hot‐spots (color spheres) important for protein engineering: red (Met200), blue (threonine triad Thr179, Thr197, Th230), green (Thr232), pink (Pro118, His119), orange (Gly206, Arg207, Asp210, Ser211). Normal font is used for observations concerning the experimental results, while italics mark inferences from molecular modeling (molecular docking, MD simulations). The original residue present at a given position is marked by white font and color circles, while the analyzed mutations are marked by black font and color circles; residues that should be present together with “Lys200” are marked with (*), while those that should be present simultaneously with “Trp200” are marked with (**). (b) Fragments of the EcAIII sequence with the role of particular residues and hints for future engineering. (c) List of previously analyzed random mutations that affected the auto‐maturation process and enzymatic activity of EcAIII (Loch et al., 2022).

The results of the nature‐inspired site‐directed mutagenesis presented in this work, as well as our previous studies with the use of random mutagenesis (Loch et al., 2022) and data available in the literature (Michalska et al., 2008) allowed us to identify the function and importance of several residues located in the area of the active site of the EcAIII molecule (Figure 7). The threonine triad, which plays a role in L‐Asn hydrolysis and in autocatalytic maturation, could be a potential target for modification. However, any manipulation of the threonine triad should be done with caution, as previous studies have shown that the T179A substitution deactivates the EcAIII enzyme by abolishing its autocatalytic maturation (Michalska et al., 2008). Better choices for substitutions in the threonine triad region seem to be Ser or Cys, as they are naturally found in other Ntn‐hydrolases (Linhorst & Lübke, 2022) or in selected orthologs (Table S3).

Our previous studies suggest that residues at positions 210 and 211, located very close to the nucleophilic Thr179, might affect the auto‐maturation process (Loch et al., 2022). In the present studies of EcAIII orthologs, we also observed the occurrence of different residues at position 211 (Table S3), which may be the result of evolutionary adaptation to ensure efficient autoproteolysis. It has been shown previously (Loch et al., 2022) that Arg207 is crucial for substrate binding, and these findings were confirmed by our simulated docking of the L‐Asn substrate to the M200W#2 structure, in which the enzyme molecule was captured in the OFF‐state and was thus unable to stabilize the substrate in the active site. This observation indicates that the highly conserved Arg207 residue must be present for efficient L‐Asn hydrolysis (and, therefore, should not be a target for mutagenesis), but the residues at positions 118, 119 and 200, in the immediate vicinity of Arg207, are more suitable for future modifications. Analysis of the data in Table S3 also shows that relatively high sequence variability is observed at position 232, although this variance is related to the type of the side chain present at position 200. Based on these observations, we have identified several hot‐spots in the EcAIII sequence that could be exploited in future engineering of EcAIII and other Class 2 L‐asparaginases, as shown in Figure 7.

## 4 CONCLUSIONS

The experimental studies combined with bioinformatic analyses performed in this work revealed that the applied mutagenesis approach was successful for EcAIII but the type of residue at position 200 had little effect on substrate affinity but affected thermal stability of EcAIII. The crystal structure of the M200L mutant confirmed that EcAIII uses the same catalytic mechanism as other Class 2 enzymes, while the crystal structure of the mutant M200W showed that Trp200 can activate a catalytic switch in the potassium‐independent EcAIII, similar to that observed in plant K‐dependent L‐asparaginases. By analyzing 25 new EcAIII orthologs from different bacterial species, we identified a number of interesting orthologs with “Lys200” or “Trp200.” These results suggest that bacterial L‐asparaginases, especially those from extremophiles and rhizosphere microorganisms, could be promising targets for future engineering and therapeutic applications. Finally, we also identified several hot‐spots in the EcAIII sequence that could be particularly useful for future enzyme engineering efforts.

## 5 MATERIALS AND METHODS

### 5.1 Sequence conservation profile

The position‐specific scoring matrix (PSSM) profile was generated using amino acid sequences of 15,600 EcAIII orthologs in bacteria that were obtained from reference (Zielezinski et al., 2022). The PSSM scores (Table S1) were calculated based on pairwise global alignments of each orthologous sequence to the reference EcAIII protein (UniProt accession: P37595, PDB: 2zal).

### 5.2 Site‐directed mutagenesis, protein expression and purification

Site‐directed mutagenesis was performed with the use of non‐overlapping primers (Table S4) according to Q5 protocol (*New England Biolabs*). As a PCR matrix, the EcAIII gene cloned to the pET11d vector was used. The presence of mutations in the EcAIII gene was confirmed by sequencing (Genomed, Poland). Large‐scale expression was performed in 1 L of liquid LB medium supplemented with 100 μg/mL ampicillin. Cells were grown at 37°C until the OD_600_ reached 1.00. The bacterial cultures were cooled to 4°C (30 min) and protein expression was induced with 0.5 mM IPTG. Protein production was carried out overnight at 18°C. Cell pellets were resuspended in a 20 mM Tris–HCl pH 8.0 buffer. Cell lysis was performed by sonication. Clarified cell lysate was loaded on an ion‐exchange column (DEAE‐Sepharose, *Merck*) connected to an ÄKTA FPLC system (*GH Healthcare*). Fractions were eluted from the column using a gradient of 2 M NaCl in 20 mM Tris–HCl pH 8.0. Fractions were analyzed by SDS‐PAGE and those containing the overexpressed protein were pooled, concentrated, and loaded on a size‐exclusion chromatography (SEC) column (Sephadex G75, *GE Healthcare*). Fractions were eluted from the SEC column using 100 mM Tris–HCl pH 8.0 with 200 mM NaCl.

### 5.3 Biophysical measurements and enzyme kinetics experiments

Prior to biophysical measurements, EcAIII variants were subjected to an additional purification step in 100 mM Tris–HCl buffer pH 8.0 with 200 mM NaCl, using a Superdex75 10/300 GL (*GE Healthcare*) column. Thermal stability was monitored by nanoDSF using a Prometheus NT.48 (*NanoTemper Technologies*) instrument. Melting scans were recorded by monitoring fluorescence emission for samples subjected to a 20–95°C temperature ramp at 1°C/min.

Kinetic parameters (*K*_M_ and *V*_max_) were determined using a modified Nessler method (Simas et al., 2021), as follows. The L‐Asn substrate concentration was in the range 0–84 mM. Reaction mixtures were incubated at room temperature (20°C) in 20 mM Tris–HCl pH 8.0 using enzymes in the concentration range 0.79–2.52 μM. Reactions were carried out in a 96‐well plate for 10 min in a final volume of 100 μL. After that time, 10 μL of each reaction mixture was transferred to a new 96‐well plate and quenched by the addition of 3 μL of 1.5 M TCA. Next, 10 μL of stabilizer solution (4 mM di‐sodium tartrate and 10 mg/L PVA) (Simas et al., 2021), 10 μL of the Nessler reagent, and 70 μL of ultrapure water were added to each well. Samples were vigorously agitated and optical density was recorded immediately at 420 nm using an Infinite M200 PRO microplate reader (*Tecan*). A calibration curve of NH_4_^+^ concentration range 0–65 mM was prepared in parallel to all reactions. Measurements were made in triplicate for enzyme assay and the calibration curve. The kinetic data were analyzed using the Michaelis–Menten model as implemented in the *Enzyme Kinetics App* of the OriginPro2020 software (OriginLab, Northampton, MA, USA).

### 5.4 Crystallization, X‐ray data collection, and structure refinement

The proteins were crystallized by vapor diffusion in a hanging drop setup at 20°C. Crystals were grown from solutions containing 20%–30% PEG4000, 10%–15% PEG400, 0.2 M MgCl_2_ (or CaCl_2_) in 100 mM Tris–HCl pH 8.5 buffer. In some cases, a crystallization additive, Gly at 1:1 protein: ligand molar ratio, was added to the crystallization drop. Co‐crystallization trials with the L‐Asn substrate were also performed, using different protein: substrate molar ratios (1:1, 1:10, 1:100). X‐Ray diffraction data were collected using synchrotron radiation from EMBL beamlines at the Petra III storage ring at DESY, Hamburg, or Cu*K*α radiation from a Synergy‐S Xtal‐LAB (*Rigaku*) home‐source generator. The diffraction images were processed using the *XDS* package (Kabsch, 2010), *Crysalis* ^*Pro*^ (*Rigaku*), and *Aimless* from the *CCP4* package (Winn et al., 2011). Structures were solved by molecular replacement using *Phaser* (McCoy et al., 2007) and refined with *REFMAC5* (Murshudov et al., 2011) using anisotropic or TLS protocols. The program *Coot* (Emsley et al., 2010) was used for model adjustment in the electron density maps and for divining solvent structure. All crystal structures were standardized in the unit cell using the *ACHESYM* server (Kowiel et al., 2014). The data collection and structure refinement statistics are summarized in Table S2. The structures were analyzed and visualized using *PyMOL* (DeLano, 2002).

### 5.5 Structural bioinformatic analyses

#### 5.5.1 Preparation of ligands for molecular docking

For docking studies, L‐Asn and L‐Asp were used as ligand molecules. Ligand preparation was performed with the LigPrep protocol implemented in the Schrödinger Suite. Tautomeric and ionization states were added using the Epik module (Greenwood et al., 2010).

#### 5.5.2 Binding site and grid generation

The grid box was placed over the binding pocket of the EcAIII/L‐Asp complex in the crystal structure with the PDB code 2zal. The grid box was generated using the Glide (Friesner et al., 2006) module.

#### 5.5.3 Molecular docking

For docking studies, the Glide software was used with the protein target rigid and the ligands flexible. The OPLS2005 (Banks et al., 2005) force field parameters were applied while performing all steps of the docking calculations. The calculated receptor‐ligand structures were scored and ranked according to the docking score of the Glide standard precision (SP) mode. For each ligand, at least five alternative poses were generated. In addition to SP mode, the Induced Fit Docking (IFD) (Sherman et al., 2006) protocol was used. This combined docking approach was based on Glide and the refinement module in Prime (Jacobson et al., 2002). This approach models conformational changes in the target protein that are induced upon ligand binding. The initial step consisted of docking each ligand using a softened potential (van der Waals radii scaling). The van der Waals scaling factor was set at 0.5 for both receptor and ligand. Then, a side‐chain orientation within a given distance of any ligand pose was predicted. At this stage, the refinement included amino acid side chains within 7 Å of the ligand. Subsequently, a minimization for each protein/ligand complex pose was performed. Finally, the ligand was docked into the receptor structure using the Glide SP mode (Halgren et al., 2004). The grid boxes for the IFD protocol were generated according to L‐Asp orientation in the PDB structure 2zal. We used the default IFD protocol, where the maximum number of poses generated per ligand is 20. Each pose of the docked L‐Asn or L‐Asp was rescored by estimation of the binding energy, using the MM‐GBSA method as implemented in the Prime (Jacobson et al., 2004) module. The value of binding energy was calculated as the difference between the energy of the protein/ligand complex and the energy of the unbound protein and ligand. The entropy term was neglected. The VSGB (Li et al., 2011) solvation model was used in the OPLS4 (Lu et al., 2021) force field with a minimized sampling method The docking simulations were carried out for a single αβ heterodimer.

#### 5.5.4 Protein structure modeling and preparation

To construct protein models of EcAIII variants and orthologs, the AlphaFold2 (Jumper et al., 2021) code was used. The models were obtained with a multimer option to construct the αβ heterodimer of each L‐asparaginase. The top‐ranked structure model was used in further analysis. The generated AlphaFold predictions and experimental X‐ray structures were prepared for molecular docking by Protein Preparation Wizard (Sastry et al., 2013). The resulting structure models were minimized using the Prime (Jacobson et al., 2002) module with the OPLS4 force field set to default convergence criteria (rms gradient value of 0.3).

#### 5.5.5 Molecular dynamics

For molecular dynamics simulation, the Desmond package (Desmond, 2021) was used. Each system was simulated at 300 K and the constant pressure of 1 bar using NPT ensemble class; a Nose‐Hoover thermostat and Martyna‐Tobias‐Klein barostat were used. The integration time step was 2 fs. The SHAKE algorithm was used to keep the hydrogen‐heavy atom bonds rigid. Smooth‐particle Ewald mesh was used for long‐range interactions and a 9 Å cutoff was set for short‐range Coulomb interactions. Before the simulation, structures were prepared and minimized. Each system was solvated in an orthorhombic box using the TIP3P water model, charges were neutralized, and 0.15 M NaCl was added. The OPLS4 force field parameters were used in all simulations. Data analysis was carried out using the Desmond tools implemented in Maestro of the Schrödinger Suite, mainly to investigate ligand interactions and protein‐ligand complex stability by monitoring rmsd and rmsf values.

## AUTHOR CONTRIBUTIONS

Maciej Janicki performed bioinformatic structural analyses (structure prediction, MD simulation, docking studies) and participated in manuscript preparation. Anna Ściuk performed site‐directed mutagenesis, protein expression purification, biochemical and biophysical studies, crystallization, X‐ray data collection, structure refinement and PDB deposition; Andrzej Zielezinski performed bioinformatic sequence analyses (multiple sequence alignments and sequence profiles) and participated in manuscript preparation; Milosz Ruszkowski performed synchrotron X‐ray data collection and processing; Agnieszka Ludwików participated in bioinformatic data analysis; Wojciech M. Karlowski participated in bioinformatic data analysis; Mariusz Jaskolski analyzed the experimental and bioinformatic data and wrote the manuscript; Joanna I. Loch participated in X‐ray data collection and processing, performed structure refinement, PDB deposition, participated in analysis of experimental and bioinformatic data, conceptualized and coordinated the work and wrote the manuscript.

## Supporting information


**Data S1.** Supporting information.

**TABLE S1.** Position‐specific scoring matrix (PSSM) of Class 2 L‐asparaginase proteins.

**TABLE S3.** Residues found at particular positions in the region of active site of Class 2 L‐asparaginases. Orthologous proteins were aligned to the reference EcAIII using global and local alignment.

**TABLE S5.** Data from MD analysis obtained for best evaluated L‐Asn docking pose.

**DATA S2.** Supporting Information.

**DATA S3.** Supporting Information.

## Data Availability

Atomic coordinates and structure factors corresponding to the final models have been deposited with the Protein Data Bank (PDB) under the accession codes: 8bqo (M200I), 8c0i (M200L), 8bkf (M200T#o), 8c23 (M200T#m), 8bi3 (M200W#1), 8bp9 (M200W#2). The corresponding raw X‐ray diffraction images have been deposited in the MX‐RDR Repository with the following Digital Object Identifiers (DOI): https://doi.org/10.18150/FYLI0H (M200I), https://doi.org/10.18150/OWO08T (M200L), https://doi.org/10.18150/ZHXXGJ (M200T#o), https://doi.org/10.18150/0CI0EE (M200T#m), https://doi.org/10.18150/KJZPRP (M200W#1), https://doi.org/10.18150/RBPJIT (M200W#2).

## Footnotes

* The modeled EcAIII orthologs have different sequence length and numbering of residues. Therefore, for clarity, in the description of amino acid changes in the modeled orthologs, we will use EcAIII numbering, with residue numbers in quotation marks. For example, “Ala232” means that an ortholog X has Ala in the position corresponding to residue 232 (Thr) of EcAIII (the original number of Ala in the sequence of X might be, of course, different). Structures of the predicted models and their sequences can be found in Supplementary data.
