# Supplementary material for: The effects of nature‐inspired amino acid substitutions on structural and biochemical properties of the E. coli L‐asparaginase EcAIII
Source: Protein Sci. 2023 Jun 1;32(6):e4647. doi: 10.1002/pro.4647 (PMC10204187; doi:10.1002/pro.4647)
Supplement: Supplementary file 1 — Data S1. Supporting information. [file PRO-32-e4647-s001.pdf]

Supplementary Tables. Table S1, **Table S3** and **Table S5** are attached as separate Excel files.

Table S2. Statistics of data collection and structure refinement. Values in parentheses correspond to the highest resolution shell.

**Structure**	**M200I**	**M200L**	**M200T#o**	**M200T#m**	**M200W#1**	**M200W#2**
**Data collection**						
Beamline/radiation source	*SuperNova*	*Synergy-S*	P13 EMBL/DESY	P13 EMBL/DESY	P13 EMBL/DESY	*Synergy-S*
Wavelength (Å)	1.54184	1.54184	0.97630	0.97630	0.97630	1.54184
Space group	$P2_12_12_1$	$P2_12_12_1$	$P2_12_12_1$	*C2*	$P2_12_12_1$	$P2_12_12_1$
Unit cell: a, b, c (Å)<br>α, β, γ (º)	52.92, 77.67, 148.28<br>90, 90, 90	49.71, 74.85,146.60<br>90, 90, 90	49.85, 75.89, 147.76<br>90, 90, 90	127.46, 149.65, 105.133<br>90, 126.72, 90	62.69, 70.84, 149.58<br>90, 90, 90	50.97, 74.48, 146.79<br>90, 90, 90
Resolution range (Å)	17.20 – 2.10<br>(2.16 – 2.10)*	19.30-1.90<br>(1.94-1.90)	73.89 – 1.22<br>(1.24 – 1.22)	84.38 – 1.84<br>(1.87 – 1.84)	74.79 – 1.45<br>(1.48 – 1.45)	73.40 – 1.70<br>(1.73 – 1.70)
Reflections collected	104865 (6750)	130791 (7634)	2114861 (97740)	936752 (45356)	1526645 (66502)	244506 (9435)
Reflections unique	35616 (2887)	42775 (2856)	166404 (8052)	133092 (6430)	117826 (5374)	61686 (3153)
Completeness (%)	97.9 (99.0)	97.6 (97.8)	99.9 (98.6)	98.0 (95.7)	99.6 (92.9)	99.1 (97.8)
Multiplicity	2.9 (2.3)	3.1 (2.7)	12.7 (12.1)	7.0 (7.1)	13.0 (12.4)	4.0 (3.1)
Wilson B-factor (Å^2)	15.10	15.50	13.60	34.90	17.40	5.30
R_{merge} (%)	14.2 (66.3)	14.8 (28.3)	7.2 (169.6)	7.1 (208.2)	8.5 (182.8)	8.2 (37.8)
R_{meas} (%)	16.9 (83.8)	17.7 (34.4)	7.5 (177.1)	7.7 (224.4)	8.8 (190.7)	9.4 (46.0)
<I/σ(I)>	5.2 (2.7)	18.0 (3.2)	16.4 (1.6)	13.9 (1.1)	16.1 (1.6)	9.9 (2.5)
$CC_{1/2}$ (%)	98.3 (51.4)	94.9 (86.5)	99.9 (59.6)	99.9 (56.4)	99.9 (79.7)	99.5 (74.9)
**Structure refinement**						
Unique / test reflections	34515 / 1064	41713 / 1022	165275 / 1018	131995 / 1088	116641 / 1068	60525 / 1056
R_{work} / R_{free} (%)	18.2 / 23.1	23.2 / 27.5	13.6 / 15.2	18.5 / 21.6	12.7 / 16.2	19.3 / 22.8
Protein / solvent atoms	4268 / 234	4227 / 185	4476 / 586	8612 / 507	4316 / 657	4356 / 267
<ADP> (Å^2) protein / solvent	19.6 / 23.5	23.13 / 25.12	18.0 / 35.0	42.19/ 46.37	24.5 / 42.3	12. 5 / 16.7
Rmsd bonds (Å) / angles (º)	0.009 / 1.52	0.010 / 1.55	0.008 / 1.45	0.013/1.63	0.009 / 1.49	0.011 / 1.60
Ramachandran plot (%)<br>favored/allowed/outliers	97 / 3 / 0	96 / 4 / 0	97 / 3 / 0	97/ 3/0	98 / 2 / 0	97 / 3 / 0
**PDB code**	**8bqo**	**8c0i**	**8bkf**	8c23	**8bi3**	**8bp9**
Diffraction data DOI	10.18150/FYLI0H	10.18150/OWO08T	10.18150/ZHXXGJ	10.18150/0CI0EE	10.18150/KJZPRP	10.18150/RBPJIT

*Values in parentheses correspond to the highest resolution shell

Table S4. Sequences (5'→ 3') of the Q5 mutagenic primers. The mutagenic codons are marked by yellow color.

mutant	Starter FOR	Starter REV
**M200I**	CACAGGCGGAATCACCAATAAATTACCC	GACGTGGCTGCCGCCAAA
**M200K**	CACAGGCGGAAAGACCAATAAATTACCC	GACGTGGCTGCCGCCAAA
**M200L**	CACAGGCGGACTGACCAATAAATTACCC	GACGTGGCTGCCGCCAAA
**M200T**	CACAGGCGGAACCACCAATAAATTACCCGGAC	GACGTGGCTGCCGCCAAA
**M200W**	CACAGGCGGATGGACCAATAAATTACCCGGAC	GACGTGGCTGCCGCCAAA

Table S6. Characteristics of bacteria species being a source of sequences of EcAIII orthologs.

species	working name	literature data	environment/habitat
**„M200I"**			
Izhakiella capsodis	***Izhakiella capsodis***	bacteria isolated from the mirid bug (*Capsodes infuscatus*) captured on summer asphodel (*Asphodelus aestivus*) plants[1]	**animal**
Bordetella B ansorpii A	***Bordetella ansorpii***	bacteria isolated from the purulent exudate of an epidermal cyst[2]	**human**
Paraburkholderia ferrariae	***Paraburkholderia ferrariae***	bacteria isolated from a high-phosphorous iron ore from Minas Gerais State (Brazil); has the ability to solubilize highly insoluble phosphatic minerals[3]	**minerals**
Caballeronia humilis	***Caballeronia humilis***	uncharacterized bacteria; bacteria from genus *Caballeronia* are part of rhizosphere and might be involved in nitrogen fixation; might promote growth of old lodgepole pine and hybrid white spruce seedlings[4]	**rhizosphere (soil)**
Vibrio sp006124995	***Vibrio sp unclassified***	uncharacterized bacteria; bacteria from genus *Vibrio* are major carbon cycle driver in marine and estuarine environments; some of them are pathogenic for animals and humans (and marine animals and plants)[5]	**water**
**„M200L"**			
Gynuella sunshinyii	***Gynuella sunshinyii***	bacteria isolated from a halophyte plant *Carex scabrifolia Steud* growing on the tidal flats at Namhae Island (South Korea); moderately halophilic and mesophilic bacterium[6]	**plant (halophyte)**
Larkinella sp004366505	***Larkinella sp unclassified***	uncharacterized bacteria; bacteria form genus *Larkinella* were isolated from the steam generator water, fermented bovine products, spruce wood decomposition and soil[7]; some species might be gamma radiation-resistant[8]	**different**
BM003 sp002868855	***Chromatiales sp unclassified***	uncharacterized bacteria; belongs to gammaproteobacteria BM003 family	**uncharacterized**
YR4-1 sp011059145	***Halalkalibacterium roseum***	bacteria isolated from a saline-alkali and sorghum-planting soil collected in Dongying (China); moderately halophilic[9]	**soil**
Schlesneria sp903904835	***Schlesneria sp unclassified***	uncharacterized bacteria; bacteria form *Schlesneria* species were isolated from acidic Sphagnum-dominated boreal wetlands of northern Russia; isolates were moderately acidophilic[10]	**water**
**„M200T"**			
UBA2146 sp002311975	***Candidatus Marinimicrobia***	uncharacterized bacteria; found at deep ocean [11,12]	**water**
Udaeobacter sp003219395	***Udaeobacter sp unclassified***	uncharacterized bacteria;	**uncharacterized**
Phenylobacterium sp013822795	***Phenylobacterium sp unclassified***	uncharacterized bacteria; *Phenylobacterium* species can metabolize xenobiotic compounds[13]; *Phenylobacterium* contains moderately thermophilic genus of bacteria isolated from subsurface aquifer[14]; nonsaline alkaline groundwater[15], human erythroleukemia cell line K562[16]; fresh water, human blood [17]; and beach soil of in Korea [18]	**variable**
Opitutus sp903877135	***Opitutus sp unclassified***	uncharacterized bacteria; some strains of family *Opitutaceae* were isolated from rice paddy soil microcosms [19], some were found in cockroach (*Shelfordella lateralis)* gut [20] or in hyporheic freshwater [21].	**variable**
Niveispirillum irakense	***Niveispirillum irakense***	isolated form roots and the rhizosphere of rice in the region of Diwaniyah (Iraq); nitrogen-fixing bacteria [22]	**rhizosphere (soil)**

**„M200K”**			
SpSt-77 sp011362935	***Chloroflexota sp unclassified***	uncharacterized bacteria; *Chloroflexota* is the phylum of phototrophic microorganisms (green nonsulfur bacteria) [23]; *Chloroflexota* cells contain bacteriochlorophylls and carotenoids [24]; found in alkaline hot spring effluents [25]	**water**
Mucilaginibacter gossypiicola	***Mucilaginibacter gossypiicola***	isolated from cotton rhizosphere soils; possess flexirubin-type pigments and is plant-growth-promoting bacteria [26]	**rhizosphere (soil)**
Pedobacter sp009765875	***Pedobacter sp unclassified***	uncharacterized bacteria; *Pedobacter* species were isolated from soils, chilled food, fish, compost, sludge, glaciers and other extreme environments; *Pedobacter* developed different mechanism of antibiotic resistance (mainly β-lactamases) that make them environmental superbugs[27]	**variable**
Microcystis aeruginosa E	***Microcystis aeruginosa***	isolated from harmful algae blooms; is the dominant harmful cyanobacteria present in harmful algae blooms; its toxic metabolites (e.g. microcystins) can be fatal to humans[28]	**water**
Myroides guanonis	***Myroides guanonis***	isolated from prehistoric guano paintings in Magura Cave (Bulgaria); psychrotolerant bacteria[29]	**minerals**
**"M200W"**			
Martelella endophytica	***Martelella endophytica***	isolated from the roots of a halophyte (*Rosa rugosa*) collected at Namhae Island (South Korea);<br>exhibit antagonistic activity against oomycete which is plant fungal pathogen[30]	**plant (halophyte)**
Moorella thermoacetica_A	***Moorella thermoacetica***	isolated from horse feces and soil;<br>acetogenic, thermophilic bacterium [31,32]	**soil**
Sagittula stellata	***Sagittula stellata***	isolated from lignin-rich pulp mill effluent;<br>lignin-transforming bacterium from a coastal environment[33]	**water**
UM-FILTER-47-13 sp002789675	***Micavibrionaceae sp unclassified***	uncharacterized bacteria;<br>belongs to genus *Micavibrio* (*Micavibrionae* family)[34]	**uncharacterized**
Martelella limonii	***Martelella limonii***	isolated from the roots of halophytes (*Limonium tetragonum*) inhabiting tidal flats of the Sacheon area (Korea)[35]	**plant (halophyte)**

Supplementary Figures

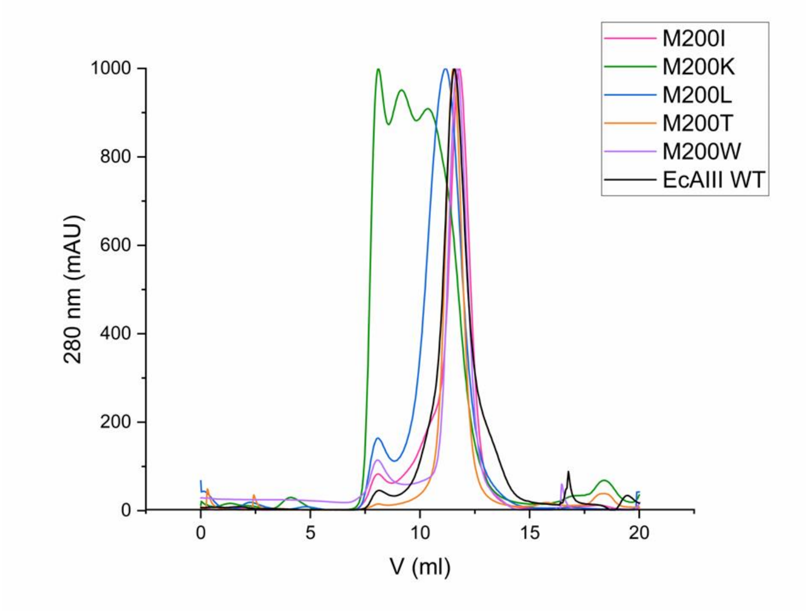

Fig. S1. Chromatograms from gel filtration experiments performed on a Sephadex G75 column.

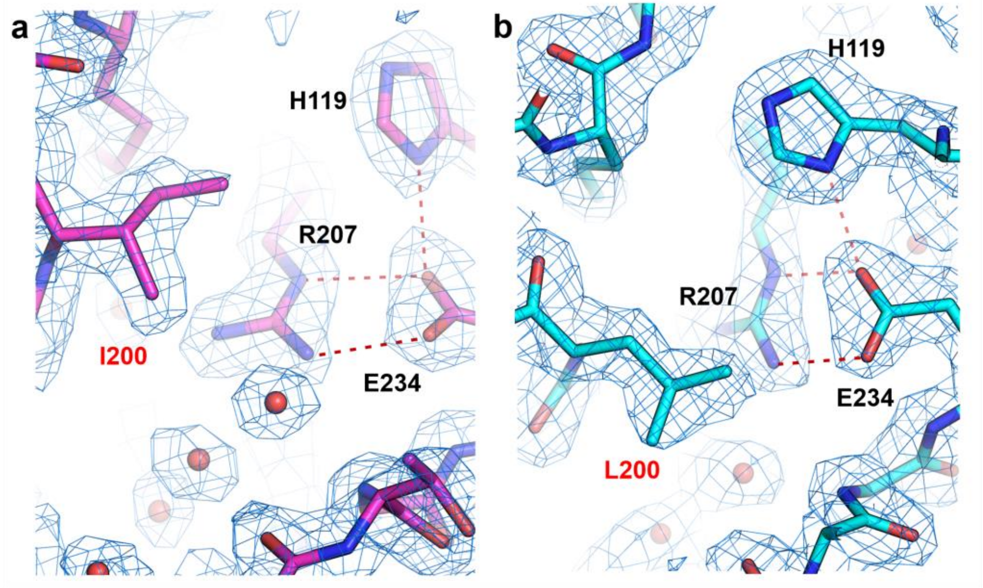

Fig. S2. 2Fo-Fc electron density maps contoured at 1.0σ around mutated residues in variants (**a**) M200I and (**b**) M200L. H-Bonds are marked as red dashed lines.

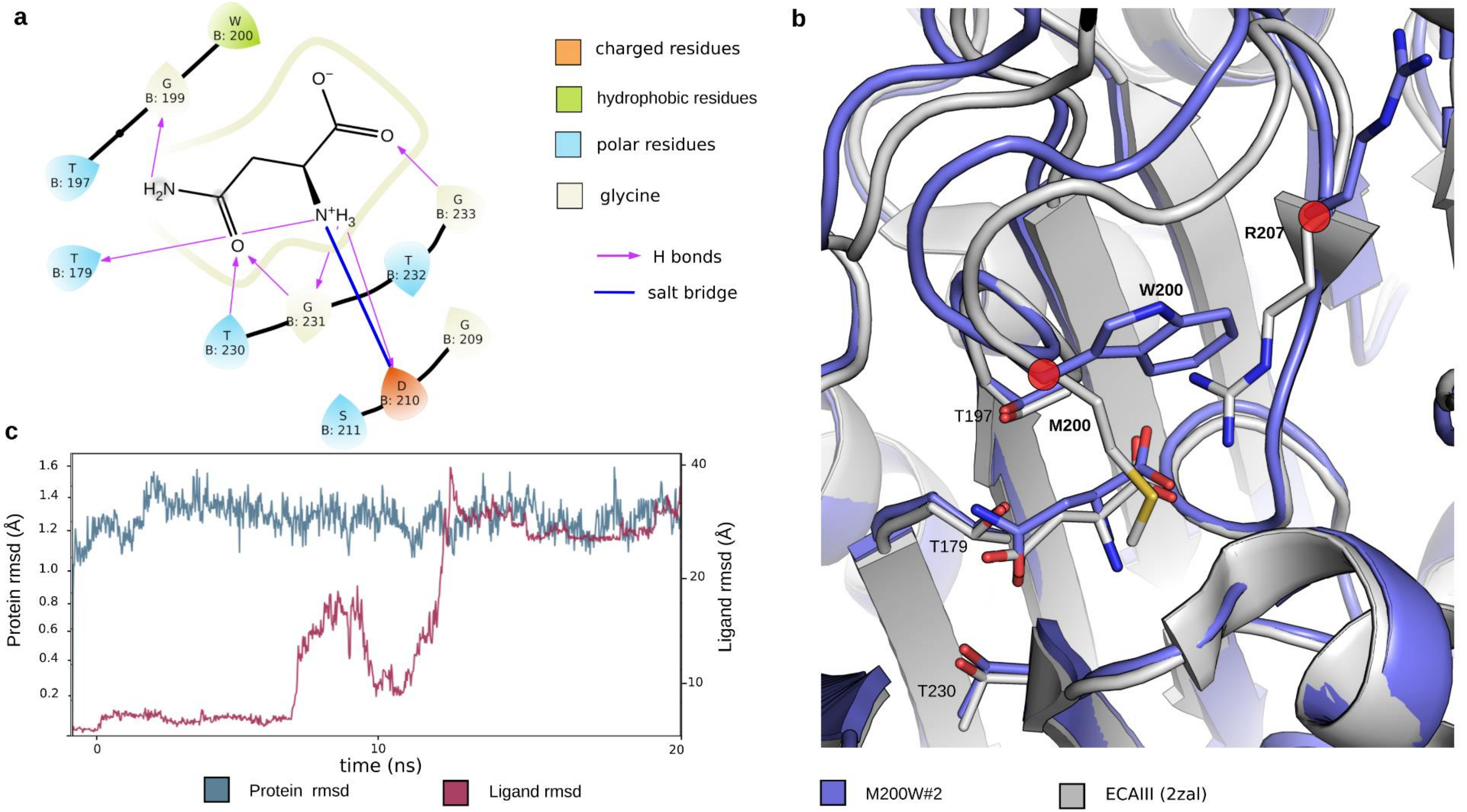

Fig. S3. Docking studies using the M200W variant as target (input structure M200W#2). (**a**) Ligand interaction diagram in complex M200W#2/L-Asn with marked interactions between L-Asn and M200W#2 variant (interacting residues marked with number and chain identifier). (**b**) Best docking pose of L-Asn, with ligand reference L-Asp from the PDB 2zal structure (shown as gray sticks), the Cα rmsd value was 0.76 Å. (**c**) rmsd plot obtained from MD for model M200W#2/L-Asn complex. The plot "protein rmsd" shows the rmsd value which was calculated based on atom selection (Cα), after all frames were aligned to the reference (first) frame. The plot "ligand rmsd" shows the rmsd value of the ligand when the protein/ligand complex is first aligned on the protein backbone of the reference (first) frame, then the rmsd of the ligand heavy atoms is measured (rmsd values significantly larger than rmsd of protein, suggest ligand diffusion from the initial binding site).

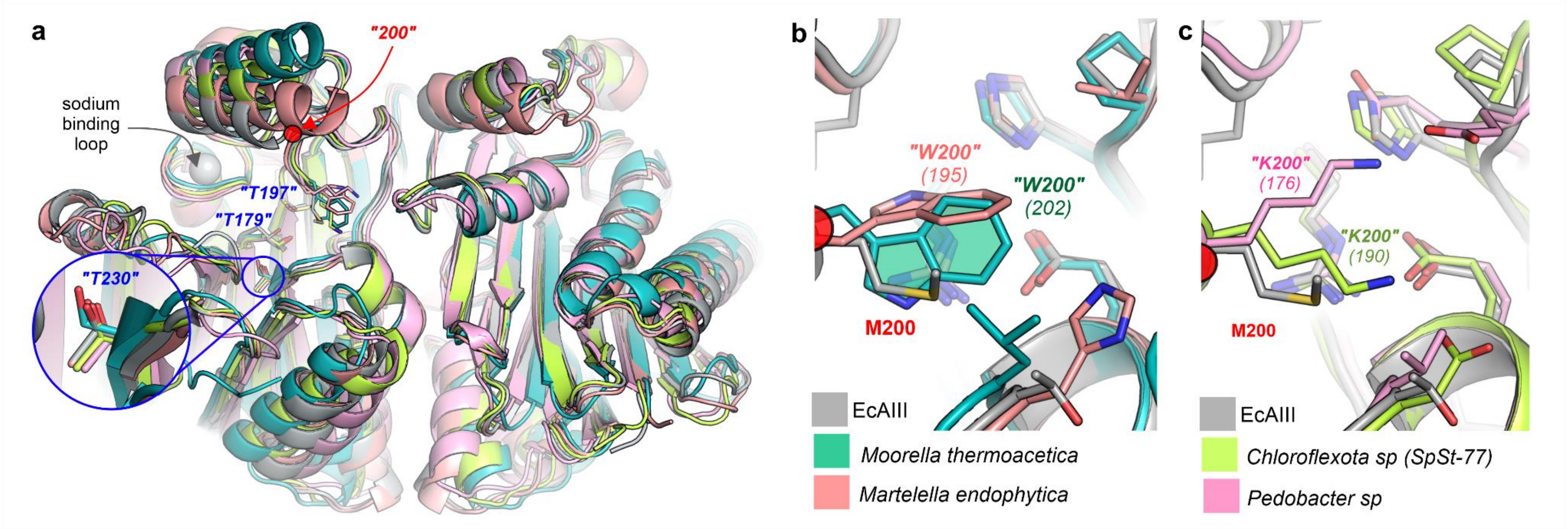

Fig. S4. (a) Superposition of the structures of selected orthologs and WT EcAIII: positions (but not always the types; blue circle) of residues from the Thr triad (blue labels) are retained, as well as the structure of the Stabilization Loop (with Na^+ shown as a gray sphere). **(b)** Alternate orientations of "Trp200" found in selected orthologs. (**c**) Alternate conformations of "Lys200" observed in the *AlphaFold2* predicted structures compared with the position of Met200 in EcAIII.

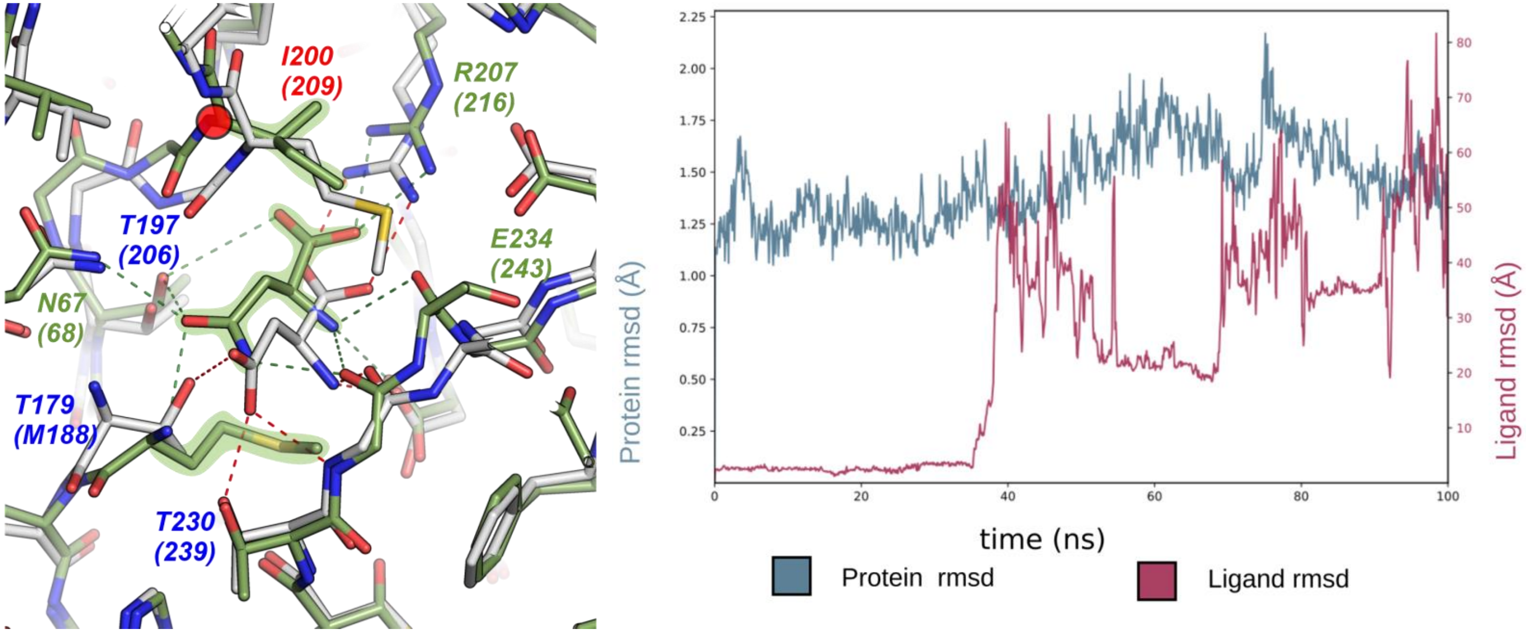

Fig. S5. Substrate docking to EcAIII ortholog from *Caballeronia humilis* (green) possessing Met instead of the nucleophilic "Thr179". The model of the protein from *C. humilis* was superposed on the crystallographic model of WT EcAIII (PDB: 2zal, gray). Red dashed lines mark H-bonds between L-Asp and WT EcAIII. Green dashed lines mark H-bonds between the *C. humilis* protein and the docked L-Asn substrate. (b) rmsd plot obtained from MD for structure model of EcAIII ortholog from *Caballeronia humilis*/L-Asn complex. The plot " protein rmsd" shows rmsd value which was calculated based on atom selection (Cα), after all frames were aligned to the reference (first) frame. The plot "ligand rmsd" shows the rmsd value of the ligand when the protein/ligand complex is first aligned on the protein backbone of the reference (first) frame, then the rmsd of the ligand heavy atoms is measured (rmsd values significantly larger than rmsd of protein, suggest ligand diffusion from the initial binding site).

Supplementary references

1. Aizenberg-Gershtein Y, Laviad S, Samuni-Blank M, Halpern M (2016) Izhakiella capsodis gen. nov., sp. nov., in the family Enterobacteriaceae, isolated from the mirid bug Capsodes infuscatus. Int. J. Syst. Evol. Microbiol. 66:1364–1370.

2. Ko KS, Peck KR, Oh WS, Lee NY, Lee JH, Song JH (2005) New species of bordetella, bordetella ansorpii sp. nov., isolated from the purulent exudate of an epidermal cyst. J. Clin. Microbiol. 43:2516–2519.

3. Valverde A, Delvasto P, Peix A, Velázquez E, Santa-Regina I, Ballester A, Rodríguez-Barrueco C, García-Balboa C, Igual JM (2006) Burkholderia ferrariae sp. nov., isolated from an iron ore in Brazil. Int. J. Syst. Evol. Microbiol. 56:2421–2425.

4. Puri A, Padda KP, Chanway CP (2020) Sustaining the growth of Pinaceae trees under nutrient-limited edaphic conditions via plant-beneficial bacteria. PLoS One 15:e0238055.

5. Grimes DJ (2020) The Vibrios: Scavengers, Symbionts, and Pathogens from the Sea. Microb. Ecol. 80:501–506.

6. Chung EJ, Park JA, Jeon CO, Chung YR (2015) Gynuella sunshinyii gen. Nov., Sp. Nov., an antifungal rhizobacterium isolated from a halophyte, carex scabrifolia steud. Int. J. Syst. Evol. Microbiol. 65:1038–1043.

7. Zhou Z, Zhu L, Dong Y, Xia X, Wu S, Wang G (2020) Larkinella punicea sp. nov., isolated from manganese mine soil. Arch. Microbiol. 202:2517–2523.

8. Park Y, Ten LN, Lee YK, Jung HY, Kim MK (2022) Larkinella humicola sp. nov., a gamma radiation-resistant bacterium isolated from soil. Arch. Microbiol. 204:1–7.

9. Wu S, Wang J, Wang J, Du X, Ran Q, Chen Q, Sheng D, Li YZ (2022) Halalkalibacterium roseum gen. nov., sp. nov., a new member of the family Balneolaceae isolated from soil. Int. J. Syst. Evol. Microbiol. 72:005339.

10. Kulichevskaya IS, Ivanova AO, Belova SE, Baulina OI, Bodelier PLE, Rijpstra WIC, Sinninghe Damsté JS, Zavarzin GA, Dedysh SN (2007) Schlesneria paludicola gen. nov., sp. nov., the first acidophilic member of the order Planctomycetales, from Sphagnum-dominated boreal wetlands. Int. J. Syst. Evol. Microbiol. 57:2680–2687.

11. Tarn J, Peoples LM, Hardy K, Cameron J, Bartlett DH (2016) Identification of Free-Living and Particle-Associated Microbial Communities Present in Hadal Regions of the Mariana Trench. Front. Microbiol. 7:665.

12. Pascoal F, Costa R, Assmy P, Duarte P, Magalhães C (2022) Exploration of the Types of Rarity in the Arctic Ocean from the Perspective of Multiple Methodologies. Microb. Ecol. 84:59–72.

13. Lingens F, Blecher R, Blecher H (1985) Phenylobacterium immobile gen. nov., sp. nov., a gram-negative bacterium that degrades the herbicide chloridazon. Int. J. Syst. Bacteriol. 35:26–39.

14. Kanso S, Patel BKC (2004) Phenylobacterium lituiforme sp. nov., a moderately thermophilic bacterium from a subsurface aquiter, and emended description of the genus Phenylobacterium. Int. J. Syst. Evol. Microbiol. 54:2141–2146.

15. Tiago I, Mendes V, Pires C, Morais P V., Veríssimo A (2005) Phenylobacterium falsum sp. nov., an Alphaproteobacterium isolated from a nonsaline alkaline groundwater, and emended description of the genus Phenylobacterium. Syst. Appl. Microbiol. 28:295–302.

16. Zhang K, Han W, Zhang R, Xu X, Pan Q, Hu X (2007) Phenylobacterium zucineum sp. nov., a facultative intracellular bacterium isolated from a human erythroleukemia cell line K562. Syst. Appl. Microbiol. 30:207–212.

17. Abraham WR, Macedo AJ, Lünsdorf H, Fischer R, Pawelczyk S, Smit J, Vancanneyt M (2008) Phylogeny by a polyphasic approach of the order Caulobacterales, proposal of Caulobacter mirabilis sp. nov., Phenylobacterium haematophilum sp. nov. and Phenylobacterium conjunctum sp. nov., and emendation of the genus Phenylobacterium. Int. J. Syst. Evol. Microbiol. 58:1939–1949.

18. Oh YS, Roh DH (2012) Phenylobacterium muchangponense sp. nov., isolated from beach soil, and emended description of the genus Phenylobacterium. Int. J. Syst. Evol. Microbiol. 62:977–983.

19. Chin KJ, Liesack W, Janssen PH (2001) Opitutus terrae gen. nov., sp. nov., to accommodate novel strains of the division "Verrucomicrobia" isolated from rice paddy soil. Int. J. Syst. Evol. Microbiol. 51:1965–1968.

20. Tegtmeier D, Belitz A, Radek R, Heimerl T, Brune A (2018) Ereboglobus luteus gen. nov. sp. nov. from cockroach guts, and new insights into the oxygen relationship of the genera Opitutus and Didymococcus (Verrucomicrobia: Opitutaceae). Syst. Appl. Microbiol. 41:101–112.

21. Baek K, Song J, Cho JC, Chung EJ, Choi A (2019) Nibricoccus aquaticus gen. nov., sp. nov., a new genus of the family Opitutaceae isolated from hyporheic freshwater. Int. J. Syst. Evol. Microbiol. 69:552–557.

22. Khammas K., Ageron E, Grimont PA., Kaiser P (1989) Azospirillum irakense sp. nov., a nitrogen-fixing bacterium associated with rice roots and rhizosphere soil. Res. Microbiol. 140:679–693.

23. Ward LM, Li-Hau F, Kakegawa T, McGlynn SE (2021) Complex History of Aerobic Respiration and Phototrophy in the Chloroflexota Class Anaerolineae Revealed by High-Quality Draft Genome of Ca. Roseilinea mizusawaensis AA3_104. Microbes Environ. 36:ME21020.

24. Gaisin VA, Kooger R, Grouzdev DS, Gorlenko VM, Pilhofer M (2020) Cryo-Electron Tomography Reveals the Complex Ultrastructural Organization of Multicellular Filamentous Chloroflexota (Chloroflexi) Bacteria. Front. Microbiol. 11:1373.

25. Madigan MT, Petersen SR, Brock TD (1974) Nutritional studies on Chloroflexus, a filamentous photosynthetic, gliding bacterium. Arch. Microbiol. 100:97–103.

26. Madhaiyan M, Poonguzhali S, Lee JS, Senthilkumar M, Lee KC, Sundaram S (2010) Mucilaginibacter gossypii sp. nov. and Mucilaginibacter gossypiicola sp. nov., plant-growth-promoting bacteria isolated from cotton rhizosphere soils. Int. J. Syst. Evol. Microbiol. 60:2451–2457.

27. Viana AT, Caetano T, Covas C, Santos T, Mendo S (2018) Environmental superbugs: The case study of Pedobacter spp. Environ. Pollut. 241:1048–1055.

28. Sun S, Tang Q, Xu H, Gao Y, Zhang W, Zhou L, Li Y, Wang J, Song C (2023) A comprehensive review on the photocatalytic inactivation of Microcystis aeruginosa: Performance, development, and mechanisms. Chemosphere 312:137239.

29. Tomova A, Tomova I, Vasileva-Tonkova E, Lazarkevich I, Stoilova-Disheva M, Lyutskanova D, Kambourova M (2013) Myroides guanonis sp. nov., isolated from prehistoric paintings. Int. J. Syst. Evol. Microbiol. 63:4266–4270.

30. Bibi F, Chung EJ, Khan A, Jeon CO, Chung YR (2013) Martelella endophytica sp. nov., an antifungal bacterium associated with a halophyte. Int. J. Syst. Evol. Microbiol. 63:2914–2919.

31. Redl S, Poehlein A, Esser C, Bengelsdorf FR, Jensen T, Jendresen CB, Tindall BJ, Daniel R, Dürre P, Nielsen AT (2020) Genome-Based Comparison of All Species of the Genus Moorella, and Status of the Species Moorella thermoacetica and Moorella thermoautotrophica. Front. Microbiol. 10:3070.

32. Drake HL, Daniel SL (2004) Physiology of the thermophilic acetogen Moorella thermoacetica. Res. Microbiol. 155:422–436.

33. Gonzalez JM, Mayer F, Moran MA, Hodson RE, Whitman WB (1997) Sagittula stellata gen. nov., sp. nov., a lignin-transforming bacterium from a coastal environment. Int. J. Syst. Bacteriol. 47:773–780.

34. Lee S, Sieradzki ET, Hazard C, Nicol GW, Lee S, Sieradzki ET, Hazard C, Nicol GW (2022) Viruses of soil ammonia oxidising archaea identified using a novel DNA stable isotope probing approach for low GC mol % genomes.

35. Chung EJ, Hwang JM, Kim KH, Jeon CO, Chung YR (2016) Martelella suaedae sp. nov. and Martelella limonii sp. nov., isolated from the root of halophytes. Int. J. Syst. Evol. Microbiol. 66:3917–3922.
